# PacBio and Illumina MiSeq Amplicon Sequencing Confirm Full Recovery of the Bacterial Community After Subacute Ruminal Acidosis Challenge in the RUSITEC System

**DOI:** 10.3389/fmicb.2020.01813

**Published:** 2020-08-07

**Authors:** Melanie Brede, Theresa Orton, Beate Pinior, Franz-Ferdinand Roch, Monika Dzieciol, Benjamin Zwirzitz, Martin Wagner, Gerhard Breves, Stefanie U. Wetzels

**Affiliations:** ^1^Institute for Physiology and Cell Biology, University of Veterinary Medicine, Hanover, Germany; ^2^Institute for Food Safety, Food Technology and Veterinary Public Health, University of Veterinary Medicine Vienna, Vienna, Austria; ^3^Austrian Competence Centre for Feed and Food Quality, Safety and Innovation FFoQSI GmbH, Tulln, Austria

**Keywords:** pH, microbiota, *in vitro*, SARA, qPCR, rumen

## Abstract

The impact of subacute rumen acidosis (SARA) on the rumen bacterial community has been frequently studied in *in vivo* trials. Here we investigated whether these alterations can be mirrored by using the rumen simulation technique (RUSITEC) as an *in vitro* model for this disease. We hypothezised that the bacterial community fully recovers after a subacute ruminal acidosis challenge. We combined a PacBio nearly full-length 16S rRNA gene analysis with 16S rRNA gene Illumina MiSeq sequencing of the V4 hypervariable region. With this hybrid approach, we aimed to get an increased taxonomic resolution of the most abundant bacterial groups and an overview of the total bacterial diversity. The experiment consisted of a control period I and a SARA challenge and ended after a control period II, of which each period lasted 5 d. Subacute acidosis was induced by applying two buffer solutions, which were reduced in their buffering capacity (SARA buffers) during the SARA challenge. Two control groups were constantly infused with the standard buffer solution. Furthermore, the two SARA buffers were combined with three different feeding variations, which differed in their concentrate-to-hay ratio. The induction of SARA led to a decrease in pH below 5.8, which then turned into a steady-state SARA. Decreasing pH values led to a reduction in bacterial diversity and richness. Moreover, the diversity of solid-associated bacteria was lower for high concentrate groups throughout all experimental periods. Generally, *Firmicutes* and *Bacteroidetes* were the predominant phyla in the solid and the liquid phase. During the SARA period, we observed a decrease in fibrolytic bacteria although lactate-producing and -utilizing families increased in certain treatment groups. The genera *Lactobacillus* and *Prevotella* dominated during the SARA period. With induction of the second control period, most bacterial groups regained their initial abundance. In conclusion, this *in vitro* model displayed typical bacterial alterations related to SARA and is capable of recovery from bouts of SARA. Therefore, this model can be used to mimic SARA under laboratory conditions and may contribute to a reduction in animal experiments.

## Introduction

The ruminal microbiota is a complex community, which enables the host animal to efficiently utilize nutrients from plant material. The microbial community is very pH sensitive and highly dependent on the diet composition. A highly energized feed ratio, which is usually fed to dairy cattle to achieve high milk yields, leads to an accumulation of short-chain fatty acids (SCFA) in the rumen and to postprandial fluctuations in ruminal pH. If the ratio between uptake and production of acids is out of equilibrium, ruminal pH decreases, and rumen acidosis may occur. Subacute rumen acidosis (SARA) is a common metabolic disorder in modern dairy production and, therefore, a major topic of interest. In recent studies, SARA has been defined as a time period of more than 330 min below pH 5.8 (Zebeli et al., 2012) or for longer than 180 min below pH 5.6 (Khafipour et al., 2009b). Subacidic conditions lead to alterations in the microbial community of the rumen, which have been frequently studied *in vivo* (Mao et al., 2013; Petri et al., 2013; Plaizier et al., 2017).

The rumen simulation technique (RUSITEC) by Czerkawski and Breckenridge (1977) is a well-established *in vitro* model for long-term observations of ruminal fermentation processes, which provides similar fermentation patterns compared to *in vivo* experiments (Martínez et al., 2010). Furthermore, a core prokaryotic community structure similar to the native ruminal microbial community in the RUSITEC model is confirmed in a study by Ziemer et al. (2000). This is supported by an *in vitro* study by Lengowski et al. (2016), which suggests a dynamic steady state after an adaption phase of more than 48 h. The establishment of a steady rumen bacterial community in the RUSITEC is also reported by Wetzels et al. (2018).

Recently, we developed an *in vitro* model for SARA by using the RUSITEC system (Maasjost et al., 2019), which mirrors *in vivo* SARA in most fermentation parameters. There is little information available concerning the microbial alterations occurring *in vitro* during and after a SARA challenge. Eger et al. (2017) investigated bacterial and archaeal community changes during and after severe acidosis in the RUSITEC by single-strand conformation polymorphism. This approach is considered a fingerprint method, which provides only a general overview of the microbial community. Traditional culture techniques underestimate the actual bacterial diversity of rumen samples (Chen et al., 2011; Petri et al., 2013). In the last decades, next-generation sequencing (NGS) techniques have gained major significance in microbial ecology (Myer et al., 2016). The Pacific Bioscience (PacBio) single-molecule real-time (SMRT) sequencing technology provides near full-length reads of the 16S rRNA gene for accurate taxonomic identification. However, the high costs impel most researchers to limit long-read sequencing to certain selected samples. In most recent studies (Poulsen et al., 2013; Duarte et al., 2017; Wetzels et al., 2018) concerning the rumen microbiome, shorter regions (V1–V3 or V3–V5) of the 16S rRNA gene were analyzed using Illumina MiSeq or Life Technologies Ion Torrent platforms. Mostly, this is done for economic reasons (Myer et al., 2016). The Illumina MiSeq sequencing method generates a great overview of the microbial diversity; however, due to the shorter read length, it has reduced phylogenetic resolution (Myer et al., 2016). Myer et al. (2016) compared the Illumina and PacBio sequencing methods in an *in vivo* trial and postulates that the full-length PacBio sequencing provides a higher phylogenetic depth and more accurate assignments.

To our knowledge, the alterations within the bacterial community during and after an *in vitro* SARA challenge have not yet been analyzed using two NGS methods in parallel. Therefore, in the present study, we combined the PacBio and Illumina MiSeq sequencing techniques to benefit from both methods. PacBio sequencing was used to gain a higher taxonomic resolution and identify specific effects within certain bacterial groups; however, as Illumina sequencing is still the commonly used method in the analysis of rumen microbiota, we additionally used pooled Illumina samples to get a better overview of general shifts at higher taxonomic levels and to increase comparability with *in vivo* data.

We hypothesized that the induction of SARA in the RUSITEC system affects the bacterial community composition similar to *in vivo* SARA trials. Moreover, we hypothesized that the bacterial community is able to recover from a SARA challenge in the RUSITEC system.

## Materials and Methods

### Ethics Statement

Two donor cows were housed in the Department for Physiology and Cell Biology at the University of Veterinary Medicine in Hanover. The animals were kept and treated according to the guidelines of the German Animal Welfare Act. The Lower Saxony State Office for Consumer Protection and Food Safety (LAVES) approved the previous fistulation of the donor cows by the experiment number AZ 33.4-42505-04-13A373.

### RUSITEC Experiment

**FIGURE 1:**
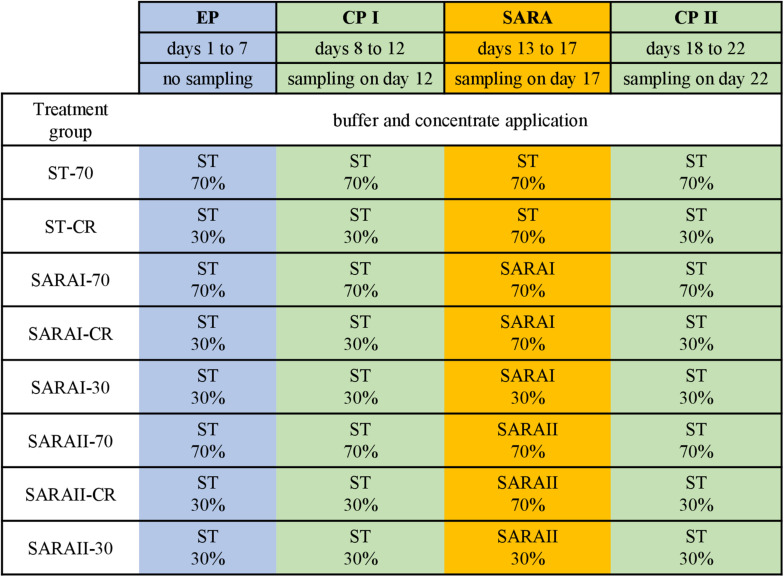
The RUSITEC experiment consisted of an equilibration period (EP, blue) of 7 days, a first control period (CP I, green) of 5 days, followed by the subacute acidosis period (SARA, 5 days, orange), and the second control period (CP II, 5 days, green). Sampling took place on the last day of CP I, SARA period, and CP II. Treatment groups are labeled as follows: ST-70 = standard buffer, 70% concentrate; ST-CR = standard buffer, changing ratio; SARAI-70 = SARA I buffer, 70% concentrate; SARAI-30 = SARA I buffer, 30% concentrate; SARAI-CR = SARA I buffer, changing ratio; SARAII-70 = SARA II buffer, 70% concentrate; SARAII-30 = SARA II buffer, 30% concentrate; SARAII-CR = SARA II buffer, changing ratio. The buffer and concentrate application for each treatment group during all experimental periods is listed as following: type of buffer (top) concentrate ratio (bottom). The type of buffer is labeled as ST = standard buffer, SARAI = SARA I buffer, SARAII = SARA II buffer.

The present study is part of an *in vitro* experiment, in which two RUSITEC systems (Czerkawski and Breckenridge, 1977) with eight fermentation vessels were used to observe long-term effects on fermentation parameters during and after a SARA challenge (Orton et al., 2020). Inoculation and daily procedures of the RUSITEC system were performed as described previously (Eger et al., 2017; Wetzels et al., 2018). The experiment consisted of four experimental runs with the same treatment groups (*n* = 4). Briefly, mixed rumen contents from both cows were used to inoculate the fermentation vessels at the beginning of the experiment. Each run started with an equilibration period of 7 d. After a first control period (CP I, days 8–12) with infusion of standard buffer (ST, Supplementary Table 1) to maintain physiological pH values, the SARA period (SARA, days 13–17) was induced by applying two different SARA buffers (SARAI and SARAII) to six out of the eight vessels. These buffers were reduced in the amount of buffering substances (Supplementary Table 1). After the SARA challenge, a second control period (CP II, days 18–22) was induced (Figure 1) by infusion of ST buffer. Two vessels served as control treatments and received the ST buffer throughout the whole experiment. Each SARA buffer induced SARA in three fermentation vessels, which were supplied with a different hay–concentrate ratio on a 70:30 basis (12.5 g per bag). Treatment groups received either continuous 70% concentrate and 30% hay (70), 30% concentrate and 70% hay (30), or a changing ratio (CR), in which only during the SARA period 70% concentrate was applied. During equilibration and control periods, the CR group received 30% concentrate. The combination of the feeding pattern and buffer variation resulted in eight treatment groups: SARAI-30, SARAI-70, SARAI-CR, SARAII-30, SARAII-70, SARAII-CR, ST-70, ST-CR (Figure 1). The combination ST-30 had to be excluded due to the limitation of eight fermentation vessels and was not expected to result in SARA conditions. Biochemical measurements are described in details in Orton et al. (2020). Briefly, pH and redox potential were measured daily before exchange of the feedbag using a pH and a redox electrode (Polyplast pH Sensors, Polyplast ORP Sensors, Hamilton Bonaduz AG, Bonaduz, Switzerland). The concentration of NH_3_-N was measured photometrically (Riede et al., 2013), and the concentration of SCFAs was measured by gas chromatography (Koch et al., 2006) using the daily effluent. Production rates of SCFA were calculated by multiplying the concentrations with the daily effluent volume. Lactate was measured using a commercial kit (D-Milchsäure (D-lactate)/L-Milchsäure (L-lactate), R-Biopharm AG, Roche, Mannheim, Germany). To determine degradation of hay and concentrate on days 11, 16, and 22, the substrates were weighted in separate nylon bags. The feed residues were dried, weighed, and incinerated; the remaining ash was again weighed and compared to an undigested standard to determine organic matter degradation.

### Sample Collection and DNA Extraction

Native samples were obtained during collection of rumen content at the start of each of the four experimental runs. Samples of the liquid and the solid phase of the RUSITEC fermenters were collected on days 12 (CP I), 17 (SARA), and 22 (CP II). Liquid samples were collected from the fermenter liquid phase prior to the exchange of the feedbags. The samples were stored at -20°C. To remove the solid associated microorganisms (SAM) from particles, feedbags were collected after the daily flushing and incubated in 100 ml prewarmed methyl cellulose solution (0.1% methyl cellulose, 0.9% NaCl, w/v) for 45 min at 39°C. Afterward, 130 ml of 4°C cold methyl cellulose solution was added, and bags were stored at 4°C for 4 h (Boguhn et al., 2013). The feedbags were then removed, and the solution was centrifuged (600 g, 5 min) to remove feed leftover. The supernatant was centrifuged at 27,000 *g* for 20 min. The supernatant was discarded, and the pellet was resuspended with sterile physiological saline solution and centrifuged again for 20 min at 27,000 *g*. The pellet was resuspended, and the samples were frozen at -20°C until further treatment. The DNA of both liquid and solid associated microorganisms was isolated using the DNeasy PowerSoil Kit (QIAGEN GmbH, Hilden, Germany) as described by the manufacturer’s protocol with 250 μl of sample material. However, instead of using the solution “C6” suggested by the kit, the DNA was eluted with 50 μl of 70°C prewarmed diethylpyrocarbonate (DEPC)-treated water.

The solid native samples were processed by a modified protocol from Kong et al. (2010). Thereby, 750 μl of 0.4 M potassium phosphate buffer was added to 250 mg of solid rumen content. The sample was homogenized in a bead beater (Bio101/FastPrep FP120 Instrument, Savant Instruments, New York, United States, 3 × 30 s, speed 4.5 m/s, samples kept on ice) and centrifuged for 10 min at 10,000 rpm (Eppendorf 5810R, Vienna, Austria). The liquid was discarded. The pellet was resuspended with 500 μl of prelysis buffer (20 mM Tris/Cl, 2 mM EDTA, 1% Triton-X 100; pH 8), and the suspension was heated at 95°C for 5 min. The supernatant was set aside for further treatment, and the pellet was resuspended with 1.2 mL of 0.4 M potassium phosphate, 100 μL of 100 mg/mL lysozyme, and 10 μL of 2.5 U/μL mutanolysin. The solution was incubated at 37°C for 30 min, and after the addition of 20 μL of 20 mg/mL proteinase K, it was incubated for 1 h at 56°C. After further bead beating (3 × 45 s, speed 4.5) and centrifugation steps (14,000 rpm, 3 min, Eppendorf 5810R, Vienna, Austria), the supernatant was pooled with the previous supernatant. Now, 205 μL of this supernatant was treated with the Power Soil Kit as described above. The presence of bacterial DNA in all samples was confirmed by 16S rRNA gene PCR with primers 27F (5′-AGA GTT TGA TCM TGG CTCAG-3′) and 1492R (5′-GGY TAC CTT GTT ACG ACT T-3′) (Weisburg et al., 1991).

### PacBio Sequencing

PacBio Single Molecule real-time full-length 16S rRNA gene sequencing was applied to all samples without pooling: 96 solid phase samples (eight treatments and three time periods, four runs), 96 liquid phase samples (eight treatments and three time periods, four runs), six native samples (three solid and three liquid, three runs), and two negative control samples (solid and liquid). Because of the longer reads obtained by the PacBio sequencing, this method was used for downstream analysis and statistical evaluation of shifts in the bacterial community. Bacteria-specific primers 27F (5′-AGRGTTYGATYMTGGCTCAG-3′) and 1492R (5′-RGYTACCTTGTTACGACTT-3′)^1^ were used. Barcodes were added during a second round of amplification with PacBio Barcoded Universal primers so that the amplicons could be multiplexed on four SMRT cells. Sequencing was carried out on a PacBio Sequel machine with 2.1 chemistry. The detailed library preparation and sequencing procedure is available online^2^. Three samples of the liquid phase (first run, CP I: ST-CR, SARAI-70, and SARAII-30) were excluded from the analysis due to low read quality after sequencing.

Accurate full-length 16S rRNA gene sequences were generated using PacBio’s single-molecule circular consensus sequencing. The circular consensus reads (ccs) were determined with a minimum predicted accuracy of 0.99 and the minimum number of passes set to three in the SMRT Link software package 5.1^3^. Bam files were converted into Fastq files via bam2fastq and demultiplexed.

### Illumina Amplicon Sequencing

For Illumina MiSeq Amplicon sequencing, samples from the four runs were pooled according to treatment, resulting in 50 samples: 24 solid phase samples (eight treatments for CP I, SARA, and CP II, respectively), 24 liquid phase samples (eight treatments for CP I, SARA, and CP II, respectively), and two native samples (solid and liquid). Additionally, three negative control samples (solid, liquid, and native) from the DNA extraction were processed together with the samples to identify contaminating bacterial reads. Illumina MiSeq sequencing of pooled samples was applied to increase comparability with already published *in vivo* data, which is, most of the time, based on Illumina sequencing and to gain a general overview of the microbial community composition. The hypervariable region 4 was targeted using the primer set 515F and 806R (Caporaso et al., 2011). Library preparation, including sample quality control, Nextera two-step PCR amplification, equimolar pooling of samples, and sequencing with a 250 bp paired-end reads protocol (V3) using an Illumina MiSeq sequencing platform (one lane) were performed by the Next Generation Sequencing facility of the Vienna Biocenter Core Facilities^4^.

### Read Processing and Data Analysis

Demultiplexed reads were processed with the software packages dada2 (version 1.9.1) (Callahan et al., 2016) and analyzed with phyloseq (version 1.25.2) (McMurdie and Holmes, 2013). Reads were trimmed for primer sequences, quality filtered, and dereplicated. Then, amplicon sequence variants (ASVs) were inferred after learning error rates. The standard filtering parameters of the dada2 pipeline were used for the Illumina data (maxN = 0 (DADA2 requires no Ns), truncQ = 2, rm.phix = TRUE, and maxEE = 2). The maxEE parameter sets the maximum number of “expected errors” allowed in a read. Afterward, forward and reverse Illumina reads were merged. The following filtering parameters were used for the PacBio data (minQ = 3, minLen = 1000, maxLen = 1600, maxN = 0, rm.phix = FALSE, maxEE = 2). Chimeras were removed using the consensus method in the removeBimeraDenovo command. Taxonomy was assigned using the SILVA nr v132 train set. The dada2 package also implements a method to make species-level assignments based on exact matching between ASVs and sequenced reference strains using the silva species assignment v132.fa.gz file. Reads from archaea, mitochondria, or chloroplasts were removed from both the PacBio and Illumina data sets. The R package decontam (version 1.0.0) (Davis et al., 2018) was applied to identify and eliminate prevalence-based contaminating reads using a threshold of 0.5. Consequently, 39 ASVs were excluded from the Illumina data set, and three ASVs were excluded from the PacBio data set. Furthermore, ASVs with less than 10 reads and a prevalence of less than three samples were excluded from the analysis. Alpha and beta diversity was calculated for PacBio and Illumina data. A proportion-based normalization was applied. Species richness and alpha diversity was displayed as “observed ASVs”, “Chao1”, “Shannon”, and “InvSimpson” indices.

### Quantitative Real-Time PCR

For quantification of bacterial 16S rRNA gene copy numbers, standard curves were constructed by using the primer set 341F (5′-CCT ACG GGA GGC AGC AG-3′) and 534R (5′-ATT ACC GCG GCT GCT GG-3′) (Muyzer et al., 1993) to amplify serial dilutions of purified PCR products from all sample types (liquid and solid phases) as recently described (Metzler-Zebeli et al., 2013). Briefly, DNA samples were assayed in duplicate in a 20-μL reaction mixture containing 9.7 μL DEPC-treated water, 2.5 μL 10 × buffer, 1 μL 2 mM MgCl2 (stock concentration 50 mM), 2.5 μL of each primer (stock concentration 2.5 μM), 0.5 μL undiluted EvaGreen fluorescent DNA stain (JenaBioscience, Jena, Germany), 1 μL of dNTP Mix (stock concentration 20 mM, 5 mM of each dATP, dCTP, dGTP, and dTTP; Thermofisher, Vienna, Austria), 0.3 μL of Platinum Taq DNA polymerase (5 U/μl; Thermo Fisher Scientific, Vienna, Austria), and 5 μL template (genomic DNA). The quantification of DNA was performed in an Mx3000P qPCR instrument (Stratagene, La Jolla, CA, United States) (software v.4.10) with an initial denaturation at 95°C for 3 min, followed by 45 cycles of 95°C for 5 s, 60°C for 20 s. To determine the specificity of the amplifications, dissociation curves after each reaction were recorded and carried out at 95°C for 1 min, followed by complete annealing at 50°C for 30 s, and a gradual increasing temperature up to 95°C. Post-run melting curves were checked for the presence of multiple peaks due to primer-dimers or non-specific amplification. Negative controls without templates were included in each qPCR reaction. The final copy numbers of total bacteria were calculated using the quantitative mean of the copy number (bacterial cell equivalents, BCE), per 250-μl liquid or 250-mg solid sample, including calculation of the DNA volume (5 μl) subjected to qPCR, the volume of extracted DNA (50 μl). Additionally, an average of four 16S rRNA gene copies per genome was taken into account when extrapolating the final copy numbers (Vetrovsky and Baldrian, 2013).

### Statistical Analysis

Means, standard deviations, and standard errors were calculated for groups and phases as well as groups and phases combined for each variable separately of the PacBio and qPCR data. Illumina data was obtained from pooled samples; therefore, no statistical analysis was applied here. The normal distribution of each data set was investigated by using the Shapiro-Wilks test. Due to non-normal distribution of the majority of data and the small sample size of the compared groups (*n* = 4), a Kruskal-Wallis rank sum test followed by a Dunn test with Bonferroni alpha-adjustment for *post hoc* analysis was applied to compare the differences between the phases within the same group and the groups within the same phase for each phylum, family, genus, species, ASVs, and 16S rRNA gene copy numbers. Significance was set at *P* < 0.05 for the Kruskal-Wallis test and at *P* < 0.025 for the Dunn post-test. In the following, the abundance is presented as mean ± SEM. Beta diversity was calculated based on the unweighted and weighted UniFrac distances and displayed as PCoA ordination plots. The phylogenetic tree used for calculation of the unweighted and weighted UniFrac distances was generated using FastTree^5^. The program Graph Pad Prism 8 (GraphPad Software, San Diego, CA, United States) was used to perform Spearman correlation analysis for bacterial abundance data and biochemical parameters as well as within biochemical parameters. Significance was set at *P* < 0.05. Phylotypes that were present in fewer than six samples were excluded from correlation analysis.

## Results

### Induction of the Subacute Rumen Acidosis

The induction of SARA led to a pH decrease below the SARA thresholds of pH 5.8 and pH 5.6 in groups treated with a SARA buffer as depicted in our partner study (Orton et al., 2020). The area under the curve for pH 5.6 was comparable to *in vivo* studies for the SARAI-30, SARAI-70, and SARAII-70 groups. With infusion of the standard buffer during CP II, pH values increased instantly.

### Illumina MiSeq Sequencing Confirms PacBio Sequencing Results

Sequencing results have been validated by combining two sequencing techniques, PacBio and Illumina MiSeq. Using the PacBio sequencing, a total of 491,134 reads were produced, ranging from 637 to 7944 reads per sample. In total, 3814 ASVs were detected. Within the 3814 ASVs, 17 phyla were detected (Supplementary Figure 1). Illumina sequencing on pooled samples was used as a standard method for rumen microorganisms. Considering all 53 Illumina samples, including two native and three negative control samples, a total of 3,936,792 processed reads was produced. Read counts ranged from 45,910 to 111,883 reads per sample. A total of 32,476 ASVs were identified, and the 50 most abundant ASVs in the liquid and solid phases are presented in Supplementary Figure 2. In total, 21 phyla were detected (Supplementary Figure 3). Although all sequences were assigned at the phylum level and more than 99% at the class and order levels in both approaches, at the family level, 90% of the PacBio sequences and 92% of the Illumina sequences were classified. In contrast, more sequences were assigned for the PacBio approach compared to Illumina at the genus (74.6 vs 71.7%) and species level (0.9 vs 0.3%). Although the absolute number of identified orders (66 vs 49), families (103 vs 65), genera (238 vs 139), and species (52 vs 25) was higher using the Illumina data set, the relative number of identified taxa at the genus and species levels was higher in the PacBio data set compared to the Illumina data set.

In both approaches, *Firmicutes* was the most relatively abundant phylum in the solid phase (Illumina: 57.9 ± 1.4%, PacBio: 48.8 ± 1.4%) and *Bacteroidetes* in the liquid phase (Illumina: 55.7 ± 2.3%, PacBio: 41.5 ± 1.1%, Supplementary Figure 4). The third most relatively abundant phylum was Proteobacteria in the Illumina approach and *Actinobacteria* in the PacBio approach. The four phyla that were not detected in the PacBio approach were minor phyla with a low overall relative abundance. At the family level, *Prevotellaceae* was the most relatively abundant family in both approaches (PacBio: 19.4 ± 0.7%, Illumina: 28.4 ± 1.9%). Furthermore, *Veillonellaceae*, *Lactobacillaceae*, *Rikenellaceae*, *Lachnospiraceae*, *Ruminococcaceae*, and *F082* were among the most relatively abundant families in both approaches. In general, the relative abundances at the phylum and family levels were comparable for both approaches (Supplementary Figure 4). Only the phylum *Actinobacteria* and its family *Bifidobacteria* were remarkably more relatively abundant in the PacBio data set. The 50 most relatively abundant genera detected in the PacBio and Illumina data sets are shown in Supplementary Table 2. Although the order and relative abundances are not the same, most highly abundant genera and ASVs detected in one data set were also among the most relatively abundant ASVs in the other data set; however, larger differences were visible for *Ruminicoccaceae* groups and *Lactobacillus* (Supplementary Figure 2 and Supplementary Table 2).

### Bacterial Richness and Alpha Diversity

**FIGURE 2:**
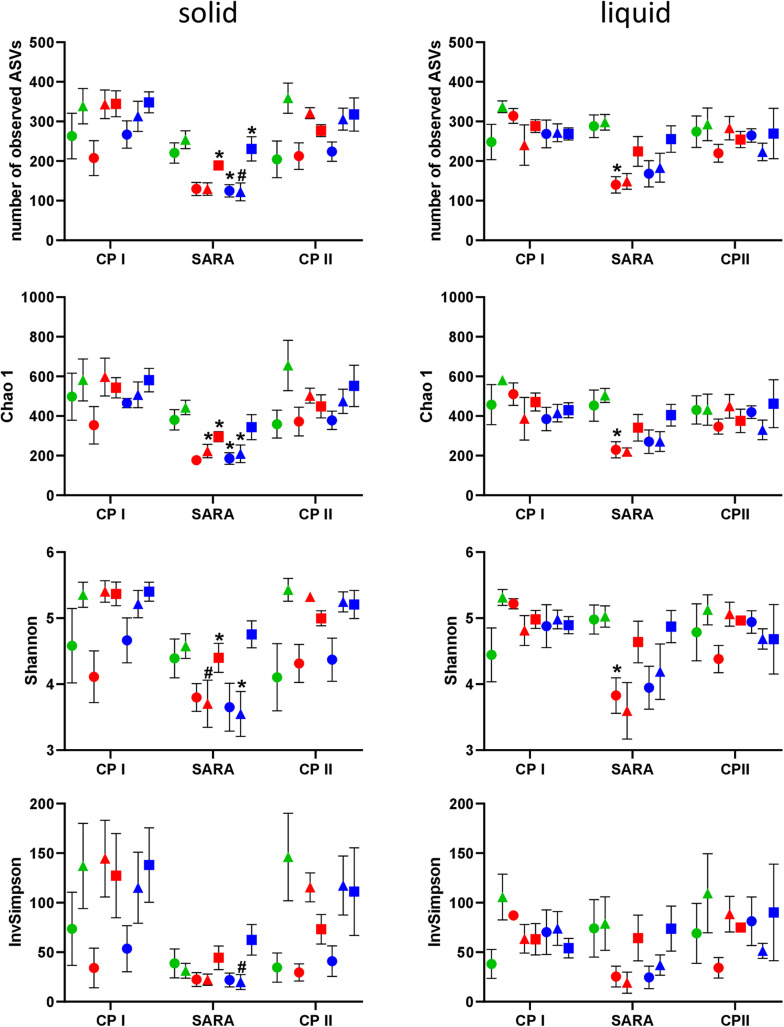
Alpha diversity of the RUSITEC samples analyzed by PacBio sequencing. Samples were collected during control period I (CP I), subacute rumen acidosis period (SARA), and control period II (CP II). Samples from the solid (left) and liquid (right) phase of the fermenters were collected. Alpha diversity measures were calculated. The treatment groups are marked according to the buffer type by colors (green = standard buffer, red = SARA I buffer, blue = SARA II buffer) and according to the concentrate ratio by shape (dots = 70% concentrate, triangles = changing ratio, squares = 30% concentrate). Asterisks indicate groups that differ significantly between CP I and SARA. Hashes indicate with significant differences between SARA and CP II. Data are presented as means ± SEM.

In the PacBio approach, the number of observed ASVs in the solid phase was significantly lower during the SARA phase for SARAI-30, SARAII-70, and SARAII-30 compared to CP I (*P* < 0.025) and compared to CP II for SARAII-CR (*P* = 0.024, Figure 2, left panel). The Chao 1 index was reduced in SARAI-CR, SARAI-30, SARAII-70, and SARAII-CR during the SARA period compared to CP I (*P* < 0.025, Figure 2, left panel). For the Shannon index, significant changes compared to either CP I or CP II were revealed for SARAI-CR, SARAI-30, and SARAII-CR, and the InvSimpson index was lower for SARAII-CR during SARA compared to CP II (*P* < 0.025, Figure 2, left panel). In the liquid phase, only the group SARAI-70 exhibited a significantly reduced number of observed ASVs, Chao 1 index, and Shannon index during the SARA period compared to CP I (*P* < 0.001, Figure 2, right panel). This also led to significant differences among groups during the SARA period in both phases’ observed ASVs, Chao 1 index, and Shannon index (Kruskall-Wallis test *P* < 0.05); however, the only difference that could be localized in the post test was for Chao 1 index between ST-CR group and SARAI-70 group for the solid phase (*P* = 0.015). In both phases, CP I and CP II did not differ significantly in alpha-diversity measures.

### Shifts in the Bacterial Community Structure Between Control and SARA Periods

**FIGURE 3:**
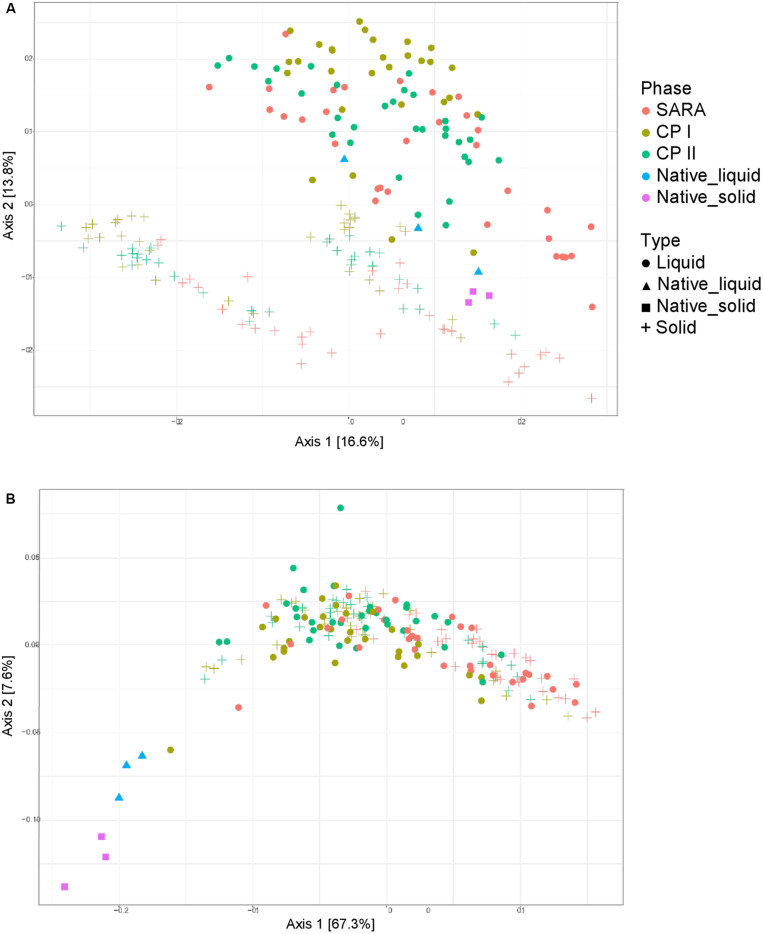
Unweighted **(A)** and weighted **(B)** UniFrac distances of samples analyzed by PacBio sequencing. Samples of control period I (CP I, colored in green) and control period II (CP II, colored in turquoise), and during SARA conditions (SARA, colored in red) are presented for the liquid (dots) and solid phase (crosses).

Unweighted UniFrac distances calculated based on the PacBio sequencing data show a separation between solid and liquid samples (Figure 3A). Samples from the SARA period tended to be located more toward the bottom right corner in the PCoA plot; however, there was no clear separation. Although the ST-buffer SARA samples were located among CP I and CP II samples, also some samples from SARA I– and SARA II–buffer treated groups were located in between the control period samples. The weighted UniFrac distances revealed a clustering of native rumen liquid and solid samples, however, no distinct pattern among RUSITEC samples (Figure 3B). Due to the lower number of sequences obtained per RUSITEC sample in the PacBio approach, the sequencing depth was apparently not sufficient to cover the whole bacterial diversity within the RUSITEC samples (Supplementary Figure 5), and therefore, alpha and beta diversity analyses have to be interpreted with caution. We, therefore, additionally performed alpha- and beta-diversity analysis based on the pooled Illumina samples.

### Alpha and Beta Diversity Assessed by Illumina Sequencing

**FIGURE 4:**
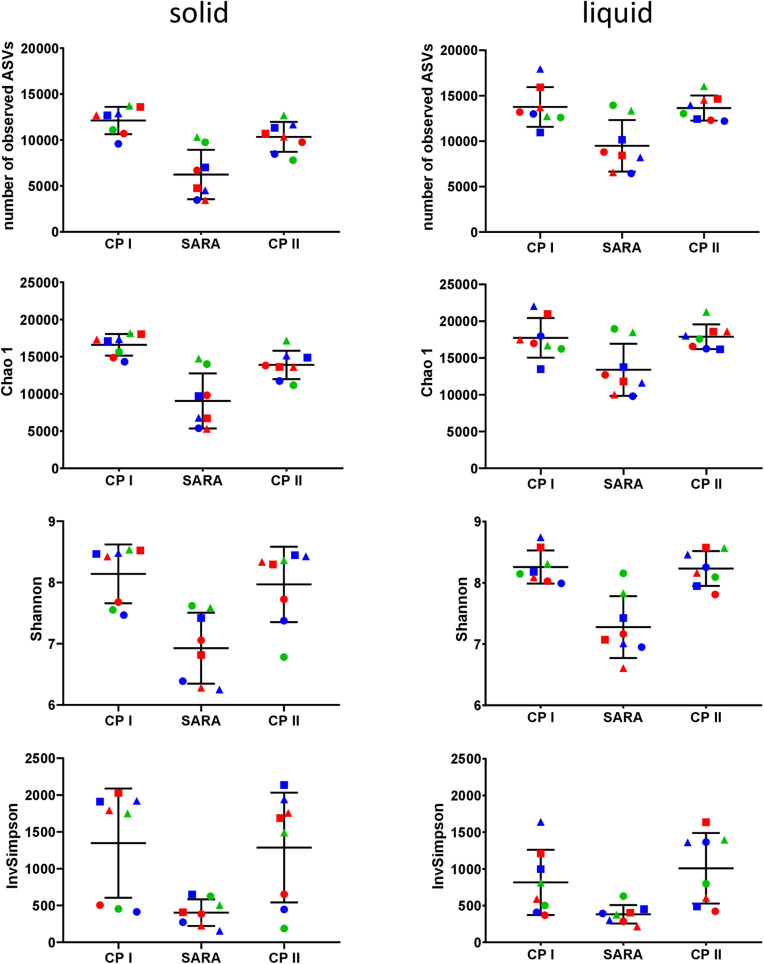
Alpha diversity of the RUSITEC samples collected during control period I (CP I), subacute rumen acidosis period (SARA) and control period II (CP II). Samples from the solid (left) and liquid (right) phase of the fermenters were collected, pooled, and subjected to Illumina MiSeq amplicon sequencing (V4 region of the 16S rRNA gene). Alpha diversity measures were calculated. The treatment groups are marked according to the buffer type by colors (green = standard buffer, red = SARA I buffer, blue = SARA II buffer) and according to the concentrate ratio by shape (dots = 70% concentrate, triangles = changing ratio, squares = 30% concentrate).

In the Illumina approach, the number of observed ASVs, the predicted richness (Chao1 index), and the Shannon diversity index decreased for all SARAI and SARAII buffer treated groups during the SARA period in both phases (Figure 4). Moreover, the InvSimpson diversity index revealed that the diversity of all three high-concentrate-fed groups in the solid phase was lower throughout all periods (Figure 4). The alpha-diversity measures did not differ between CP I and CP II. In the liquid phase, control groups remained stable throughout the whole experiment.

**FIGURE 5:**
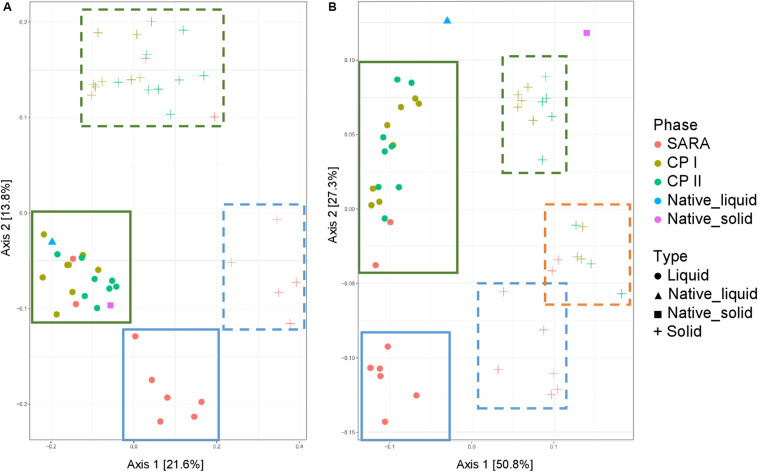
Unweighted **(A)** and weighted **(B)** UniFrac distances of samples analyzed by Illumina MiSeq (V4) imply a clustering of treatment groups during control period I (CP I, colored in green) and control period II (CP II, colored in turquoise), and during SARA conditions (SARA, colored in red) for the liquid (dots) and solid phase (crosses). Clusters in the liquid phase are marked with boxes with solid lines, clusters within the solid phase are marked by boxes with broken lines.

Both the unweighted (Figure 5A) and weighted (Figure 5B) UniFrac distances for the liquid samples, six out of the eight treatment groups clustered together during the SARA challenge (Figure 5, blue boxes), and a clear shift was visible compared to CP I and CP II (Figure 5, green boxes). These clusters respresented the SARA buffer–treated groups (Supplementary Figures 6C,D). In contrast, the two ST buffer samples of the SARA period clustered together with the samples from CP I and CP II (Figure 5, green boxes, Supplementary Figures 6C,D). A similar pattern was observed for the solid phase samples in the unweighted UniFrac analysis (Figure 5A, blue box, broken line) with one additional SARA sample located close to the CP samples (Figure 5A, green boxes, broken line). In the weighted analysis, three clusters appeared to be present for the solid samples, one in the bottom containing most SARA period samples (Figure 5B, blue box, broken line), one in the middle containing the two ST buffer SARA period samples and CP samples from the high-concentrate groups (Figure 5B, orange box, broken line, Supplementary Figure 6B), and one in the top of the graph containing low-concentrate and changing ratio CP samples (Figure 5B, green box, broken line, Supplementary Figure 6B). The native rumen samples clustered together with CP I and CP II samples in the unweighted phase and separately for the weighted UniFrac analysis (Figure 5).

### Alterations in the Relative Abundance of Bacterial Phyla and Families Detected by PacBio Sequencing

The three main phyla *Firmicutes*, *Bacteroidetes*, and *Actinobacteria* were not significantly altered during the experiment. However, the less relatively abundant phyla *Fibrobacteres*, *Lentisphaerae, Kiritimatiellaeota*, *Planctomycetes*, *Spirochaetes*, *Tenericutes*, and *Verrucomicrobia* exhibited significantly reduced relative abundances in several SARA-buffer treated groups during the SARA challenge in both phases (at least *P* < 0.05, Supplementary Figure 1, Supplementary Table 3). The only phylum that decreased during the SARA challenge and did not recover for all groups during CP II was *Elusimicrobia*.

**FIGURE 6:**
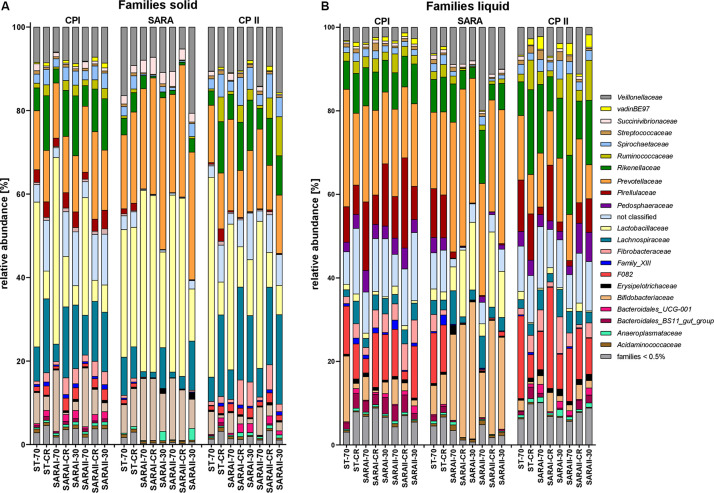
Relative abundances on family level detected by the PacBio amplicon sequencing approach for the solid **(A)** and liquid **(B)** phases. Families with an abundance of less than 0.5% were combined into one group. Relative abundances for each treatment group are shown for all three experimental periods (CP I = control period I, SARA = subacute acidosis period, CP II = control period II). Treatment groups are labeled as follows: ST-70 = standard buffer, 70% concentrate; ST-CR = standard buffer, changing ratio; SARAI-70 = SARA I buffer, 70% concentrate; SARAI-30 = SARA I buffer, 30% concentrate; SARAI-CR = SARA I buffer, changing ratio; SARAII-70 = SARA II buffer, 70% concentrate; SARAII-30 = SARA II buffer, 30% concentrate; SARAII-CR = SARA II buffer, changing ratio.

The most relatively abundant families in the solid phase were *Lactobacillaceae* (23.5 ± 2.1%) and *Prevotellaceae* (18.5 ± 1.0%, Figure 6A), and in the liquid phase *Prevotellaceae* (20.3 ± 1.1%) dominated followed by *Rikenellaceae* (9.6 ± 0.6%, Figure 6B). Family *Lactobacillaceae* was significantly enriched during SARA compared to either CP I or CP II in three treatment groups in the solid phase and in four groups in the liquid phase (at least *P* < 0.05, Figure 6, Supplementary Table 4). These groups included SARA-buffer treated groups and the ST-CR treatment. Moreover, the relative abundance of *Prevotellaceae* was higher in the SARA challenge compared to CP II in several groups in both phases (at least *P* < 0.05, Figure 6, Supplementary Table 4). In contrast, the family *Veillonellaceae* only increased during SARA in a few groups in the liquid phase (*P* < 0.05, Figure 6, Supplementary Table 4). The family *Fibrobacteriaceae* was reduced during the SARA period in all SARA-buffer treated groups (at least *P* < 0.05, Figure 6, Supplementary Table 4). Moreover, the families *Rikenellaceae*, *Ruminococcaceae*, *Pirellulaceae*, and *Spirochaetaceae* declined during the SARA period in several treatments in both phases (at least *P* < 0.05, Figure 6, Supplementary Table 4). The family *Lachnospiraceae* was only affected in the solid phase for SARA compared to CP II (at least *P* < 0.05, Figure 6, Supplementary Table 4). In the ST-70 group, none of the families changed significantly. In contrast, the ST-CR exhibited significant changes for *Endomicrobiacea*e, *Lachnospiraceae*, *Lactobacillaceae*, and *Rikenellaceae* (at least *P* < 0.05, Figure 6, Supplementary Table 4). Most of the SARA-related changes were also transient on the family level (Figure 6, Supplementary Table 4). All families with significant changes and with a mean relative abundance >0.5% are listed in Supplementary Table 4.

### Changes in the Bacterial Community Composition on Genus and Species Level Detected by PacBio Sequencing

**FIGURE 7:**
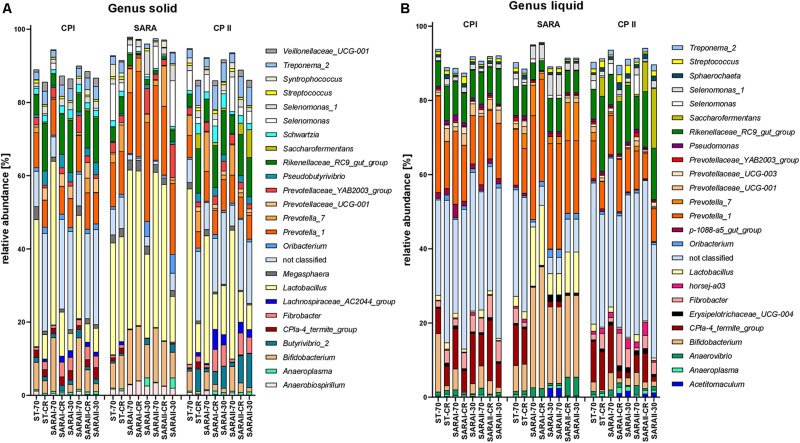
Relative abundances on genus level detected by the PacBio amplicon sequencing approach for the solid **(A)** and liquid **(B)** phases. Only the 25 most abundant genera of each phase are displayed. Relative abundances for each treatment group are shown for all three experimental periods (CP I = control period I, SARA = subacute acidosis period, CP II = control period II). Treatment groups are labeled as follows: ST-70 = standard buffer, 70% concentrate; ST-CR = standard buffer, changing ratio; SARAI-70 = SARA I buffer, 70% concentrate; SARAI-30 = SARA I buffer, 30% concentrate; SARAI-CR = SARA I buffer, changing ratio; SARAII-70 = SARA II buffer, 70% concentrate; SARAII-30 = SARA II buffer, 30% concentrate; SARAII-CR = SARA II buffer, changing ratio.

The 25 most relatively abundant genera in the solid and liquid phases are presented in Figure 7. In the solid phase, *Lactobacillus* was the most abundant genus among all samples (24.0 ± 1.6%); in the liquid phase, unclassified genera dominated (26.8 ± 2.1%), followed by the group *Prevotella 1* (16.4 ± 1.1%). Significant changes were also identified for the 25 most relatively abundant genera in each phase (Supplementary Table 5). In the liquid phase, the genera *Anaeroplasma*, *Erisypelotrichaceae UCG-004*, *horsej-a03*, *p-1088-a5 gut group*, *Pseudomonas*, and *Sphaerochaeta* were reduced during the SARA period compared to either CP I or CP II for several SARA-buffer treated groups. In the solid phase, the genera *Butyrivibrio 2*, *Lachnospiraceae AC2004 group*, *Pseudobutyrivibrio*, *Schwarztia*, and *Veillonellaceae UCG-001* were less relatively abundant during the SARA period in several groups infused with SARAI or SARAII buffer, and *Oribacterium* was more abundant during SARA in two groups (SARAI-30, SARAII-30) and *Selenomonas 1* in one group (SARAI-30). The *CPla-4 termite group* decreased in several groups in both phases; however, it also differed between CP I and CP II for three groups. A decrease in relative abundance during SARA induction was also observed in both phases for *Fibrobacter*, *Rikenellaceae RC9 gut group*, and *Treponema 2* for several groups. In contrast, the relative abundance of the genus *Lactobacillu*s was elevated during the SARA phase in two groups in the solid phase and in three groups in the liquid phase. Within the family *Prevotellaceae*, the five analyzed genera groups behaved differently. The groups *Prevotella 1*, *Prevotella 7*, and *Prevotellaceae YAB2003* increased in the solid phase during SARA in some of the SARA-buffer treated groups. In contrast, *Prevotellaceae UCG-001* decreased in most groups in the solid phase and in two groups in the liquid phase and *Prevotellaceae UCG-003* decreased for three SARA-buffer groups in the liquid phase. Six genera exhibited also alterations by the diet change in ST-CR group, and the ST-70 group only differed for *horsej-a03* between CP I and CP II.

Of the 25 identified species, few species were significantly altered due to SARA induction. In the solid phase, *Lactobacillus mucosae* increased significantly for ST-CR (*P* = 0.016 compared to CP II), and *Pseudobutyrivibro ruminis* was significantly reduced during SARA for SARAI-CR and SARAII-CR (both *P* = 0.012) compared to CP II and differed between CP I and CP II for ST-CR (both *P* = 0.016) and SARAI-30 (data not shown). Moreover, *Acidaminococcus fermentans* was enriched during SARA in SARAII-CR. *Kandleria vitulina* and *Succinivibrio dexinosolvens* differed between CP I and CP II in one treatment group each (at least *P* < 0.025). In the liquid phase, *Lactobacillus mucosae* was significantly enriched in the SARAI-70 and SARAII-CR groups during the SARA period and *Lactobacillus amylovorus* was enriched in the SARAI-70 and SARAI-CR groups compared to one of the control periods (at least *P* < 0.016). Moreover, *Pseudomonas formosensis* was reduced during in the SARAI-70 treatment (*P* = 0.018, compared to CP I).

### Alterations of the Most Abundant ASVs Detected by the PacBio Sequencing

**FIGURE 8:**
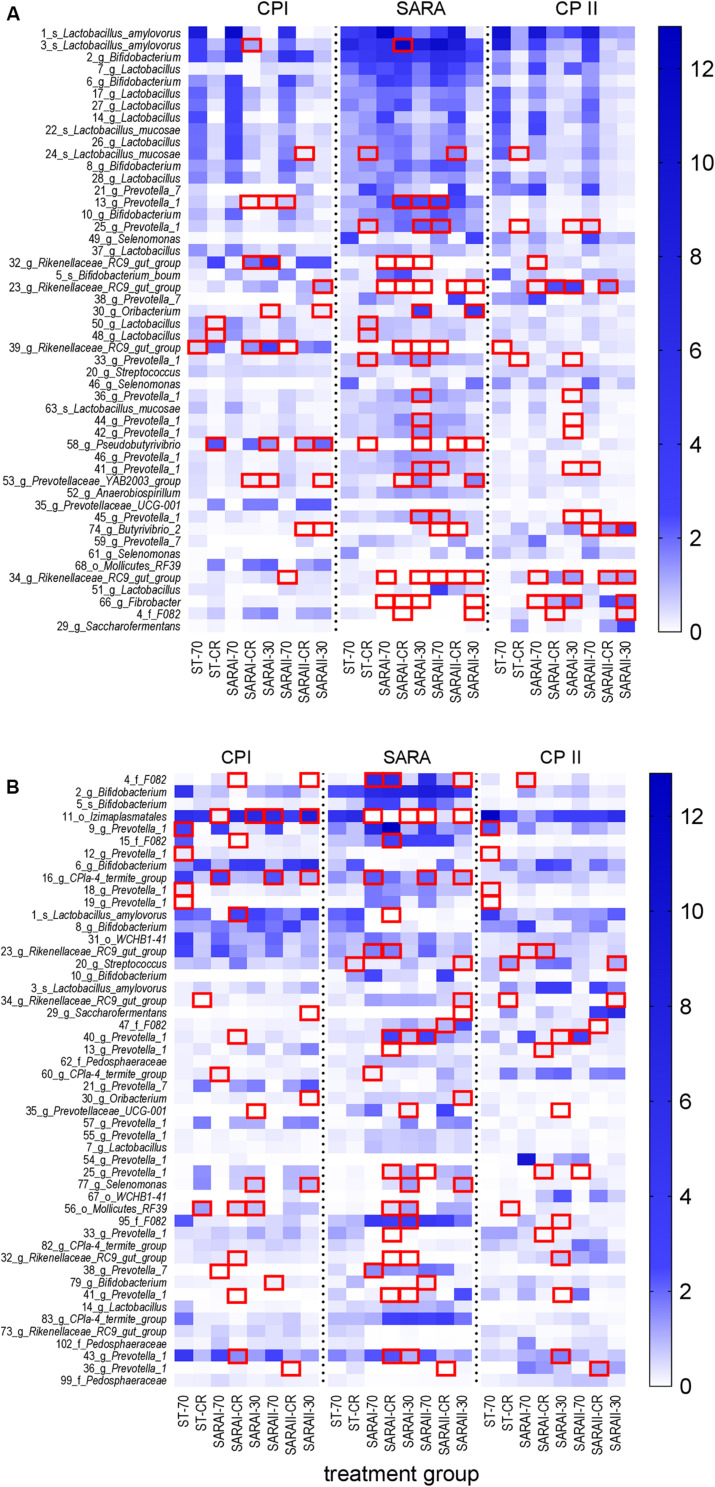
Alterations of the 50 most abundant amplicon sequence variants (ASV) in the PacBio analysis throughout all experimental periods (left: CP I = control period I, middle: AP = acidosis period, right: CP II = control period II) for the solid **(A)** and liquid **(B)** phases. ASVs are labeled with the ASV-number, followed by the lowest classification level (*o* = order, *f* = family, *g* = genus, *s* = species) and the taxonomic identification. Samples of all four runs were statistically analyzed and are labeled as follows: ST-70 = standard buffer, 70% concentrate; ST-CR = standard buffer, changing ratio; SARAI-70 = SARA I buffer, 70% concentrate; SARAI-30 = SARA I buffer, 30% concentrate; SARAI-CR = SARA I buffer, changing ratio; SARAII-70 = SARA II buffer, 70% concentrate; SARAII-30 = SARA II buffer, 30% concentrate; SARAII-CR = SARA II buffer, changing ratio. The relative abundance (%) is indicated by the color scale. Significant changes of ASVs throughout the three experimental periods are marked with a red square.

In the solid phase, 15 out of the 50 most abundant ASVs (Figure 8A) were associated with the genus *Lactobacillus*, of which five were classified at the species level (*Lactobacillus amylovorus* and *Lactobacillus mucosae*). Nine ASVs were classified within the group *Prevotella 1*. Significant changes were revealed for four of these *Lactobacillus* ASVs and eight ASVs from group *Prevotella 1* (Figure 8, Supplementary Table 6). These ASVs were enriched in at least one group in the SARA period. In contrast, four ASVs from the genus *Rikenellaceae RC9 gut group* and one from genus *Fibrobacter* were reduced during the SARA challenge in several SARA-buffer treated groups (*P* < 0.05, Figure 8A, Supplementary Table 6). Most of these differences could either be detected between CP I and the SARA challenge or between the SARA challenge and CP II.

In the liquid phase, 14 ASVs from the group *Prevotella 1* dominated the 50 most relatively abundant ASVs, followed by six ASVs from genus *Bifidobacterium* (Figure 8B). In the liquid phase, most ASVs exhibited only one or two significant changes and only ASV 11 (order *Izimaplasmatales*) was significantly reduced during SARA in four SARA buffer groups (*P* < 0.05, Figure 8B, Supplementary Table 7). One ASV from genus *Selenomonas* was enriched during SARA period in the SARAI-30 and SARAII-30 groups (*P* < 0.05). Eleven ASVs from the group *Prevotella 1* changed significantly but with an inconsistent pattern: some were reduced during SARA, some enhanced, and four displayed only significant differences between CP I and CP II in the ST-70 group (Figure 8B, Supplementary Table 7).

Generally, very few changes were observed between CP I and CP II in both phases at the ASV level (Supplementary Tables 6, 7).

### Associations of Bacterial Abundances With Fermentation Parameters

**FIGURE 9:**
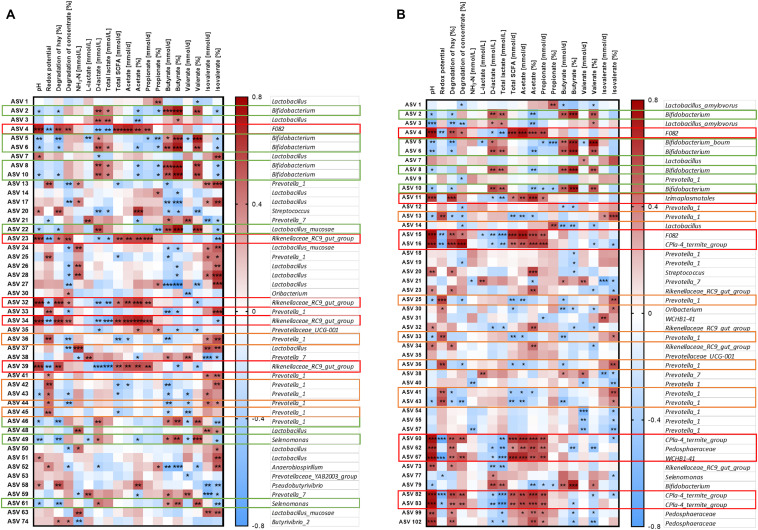
Correlation analysis of the 50 most abundant ASVs of the solid **(A)** and the liquid phase **(B)** with fermentation parameters during the SARA challenge (day 17). Spearman ρ is indicated by the red to blue color scheme. Significant correlations are indicated by *P < 0.05, **P < 0.01, ***P < 0.001. Boxes with the same color indicate similar patterns of correlations for these ASVs. ASVs that were not represented in at least six of the samples were excluded from this analysis.

The relative abundance of the 50 most relatively abundant ASVs was correlated to fermentation parameters (Orton et al., 2020) to identify functional groups. At day 17, we observed three major patterns for the ASVs. Four ASVs in the solid phase and eight ASVs in the liquid phase displayed a strong positive correlation with pH (*P* < 0.001, Figure 9, red boxes). These ASVs mainly belonged to families *F082*, *Rikenellaceae RC9 gut group*, and *CPla4 termite group.* Moreover, ASV 4 (family *F082*) was positively correlated with pH in both phases. The pH was positively correlated to the degradation of hay and the production rates of total SCFA, acetate, and propionate and the molar proportion of acetate and negatively correlated to redox potential, D-lactate, and total lactate (*P* < 0.01, Supplementary Figure 7). Therefore, these 13 ASVs were also frequently correlated to these parameters. In total, eight ASVs from group *Prevotella 1* exhibited a contrary pattern (Figure 9, orange boxes). Moreover, nine ASVs mostly belonging to the genera *Bifidobacterium* and *Selenomonas* were negatively correlated with pH and positively correlated with D-lactate, total lactate, butyrate production, butyrate proportion, and valerate proportion (Figure 9, green boxes).

### Total Abundance of Bacteria

The total number of bacteria was estimated by measuring the copy number of bacterial 16S rRNA genes. In the liquid phase, the bacterial cell equivalents were neither affected by the experimental period, nor by the treatment group. Bacterial cell equivalents per μl in the liquid phase samples ranged between 2.22 × 10^8^ ± 1.23 × 10^8^ and 1.72 × 10^9^ ± 2.20 × 10^9^. In the solid phase, three treatments (SARAI-70, SARAI-CR, SARAII-70) displayed significant alterations in the gene copy number per 250 mg during the three experimental phases with the lowest values during the SARA period (Table 1).

**TABLE 1:** Mean copy numbers of the bacterial 16S rRNA gene in solid samples.

	Bacterial cell equivalent (10^9^/g) per period^2^			pooled SD^5^ (10^9^)	*P*-value^4^ (Time)
Treatment^1^	CP I	SARA	CP II		
SARAI-70	13.9	4.4	13.1	4.2	0.025
SARAI-30	16.0	5.3	13.7	6.7	n.s.^3^
SARAI-CR	18.0^a^	3.9^b^	14.5^ab^	2.5	0.015^4^
SARAII-70	15.9^ab^	5.1^a^	13.5^b^	7.3	0.023^4^
SARAII-30	15.9	5.8	12.0	5.0	n.s.
SARAII-CR	12.8	5.8	9.3	5.3	n.s.
ST-CR	13.9	9.1	10.1	6.3	n.s.
ST-70	15.8	10.8	12.3	6.5	n.s.
*P*-value (Treatment)	n.s.	*n*.*s*.	*n*.*s*.	n.s.	n.s.

*^1^Treatment groups: SARAI-70 = SARA I buffer, 70% concentrate; SARAI-30 = SARA I buffer, 30%; SARAI-CR = SARA I buffer, changing ratio; SARAII-70 = SARA II buffer, 70% concentrate; SARAII-30 = SARA II buffer, 30%; SARAII-CR = SARA II buffer, changing ratio; ST-CR = Standard buffer, changing ratio; ST-70 = Standard buffer, 70% concentrate. ^2^period: CP I = control period I; SARA = SARA period; CP II = control period II. ^3^n.s. = not significant, *p* > 0.05. ^4^Different superscripts indicate significantly different values within one row. ^5^SD = standard deviation.*

## Discussion

The bacterial community structure of the rumen has been a major topic of interest during the past decades. It has often been demonstrated that a reduction in pH leads to a decrease in the bacterial diversity *in vivo* (Mao et al., 2013; McCann et al., 2016; Neubauer et al., 2019) as much as in *in vitro* approaches (Mickdam et al., 2016; Eger et al., 2017). Using PacBio sequencing, several treatment groups exhibited reduced diversity during SARA induction, especially in the solid phase. However, no clear clustering among samples was observed. The rarefaction analysis indicated that the sequencing depth was not sufficient to cover the whole bacterial diversity of bacteria in RUSITEC samples. Myer et al. (2016) estimated that about 40,000 reads per sample are needed to cover the whole diversity of rumen bacteria. Although bacterial diversity may be slightly lower in the RUSITEC system, read counts were much lower in the present study and the loss of low abundant species impairs significance of alpha- and beta-diversity analysis. In contrast, Illumina MiSeq of the V4 hypervariable region can reach 93% of coverage at 22,000 sequences per sample (Derakhshani et al., 2017), which was easily achieved in the present study; therefore, alpha and beta diversities were additionally analyzed based on the pooled Illumina samples. With this approach, we observed reduced bacterial richness and diversity (Chao 1, Shannon, and InvSimpson’s index) during SARA in all SARA-buffer treated groups, which is in line with the PacBio results. Moreover, samples experiencing the SARA challenge clearly clustered separately from samples with physiological pH values. This has also been demonstrated for particle- and fluid-associated microorganisms *in vivo* when SARA was induced with a grain-based concentrate (Plaizier et al., 2017; Neubauer et al., 2019); however, in the study by Plaizier et al. (2017), the effects were more pronounced in the unweighted UniFrac analysis, indicating that minor groups are more affected.

In our study, the main phyla *Bacteroidetes* and *Firmicutes* were not altered by the SARA challenge. This is in contrast to some previous studies, which report a decrease in the relative abundance of the Gram-negative phylum *Bacteroidetes* during SARA challenges (Khafipour et al., 2009b; Mao et al., 2013). However, these changes are inconsistent and also dependent on the type of feed (Plaizier et al., 2017). The destruction of Gram-negative bacteria results in increased LPS levels in the rumen, which are thought to induce systemic inflammatory conditions (Gozho et al., 2005); however, ruminal and blood LPS levels are not consistently linked among studies (Khafipour et al., 2009a; Guo et al., 2017). At a lower phylogenetic level, we observed that the relative abundance of members of the *Rikenellaceae RC9 gut group* decreased during SARA although the abundance of some *Prevotellaceae*, especially *Prevotella 1*, highly increased. *Prevotella 1* has been identified as a major genus of rumen *Prevotellaceae* and contains species, such as *Prevotella ruminicola* (Henderson et al., 2019). A suppression of *Rikenellaceae* and an increase of *Prevotellaceae* by starch-rich diets and in SARA conditions have been reported previously *in vivo* (Petri et al., 2013; Zened et al., 2013). However, a high taxonomic resolution is essential to understand the shift within certain bacterial groups as, even within one genus, different strains might have varying metabolic functions. Nevertheless, we could confirm that the change in the abundance of *Prevotella* is associated with the change in pH (Petri et al., 2013).

During the SARA challenge, *Lactobacillus* increased in SARA buffer–treated groups and the ST-CR group during the SARA challenge. Lactate-producing *Lactobacillaceae* are known to tolerate low pH values better than other rumen bacteria (Mackie and Gilchrist, 1979). Presumably, in addition to low pH values, the enhanced starch availability supports the growth of *Lactobacillaceae*. Previously, Yang et al. (2018) also reported a high colonization of grain by *Lactobacilli* in cattle without SARA and hypothesized that they play a common role in starch degradation. The increasing abundance of the lactate-producing *Lactobacillaceae* was accompanied by an enhanced abundance of the Gram-negative family *Veillonellaceae* within the liquid phase. Members of this family, such as *Megasphaera elsdenii*, tolerate pH values in the range of SARA (Russell and Dombrowski, 1980) and utilize lactate for SCFA production (Chen et al., 2019). The parallel growth of lactate-producing bacteria and lactate-utilizers might contribute to the low lactate levels, which were observed in this study (Orton et al., 2020) and which are also typical for SARA *in vivo* (Krause et al., 2009).

During the SARA challenge, we also observed a decrease in the relative abundance of *Fibrobacteres* (*Fibrobacter*), *Ruminococcaceae*, *Lentisphaeare*, *Spirochaetes* (*Treponema* and *Sphaerochaeta*), and a few other taxa. Members of *Fibrobacter* and the *Ruminococcaceae* are important cellulolytic bacteria in the rumen (Berg Miller et al., 2009; Suen et al., 2011). During SARA conditions, these cellulolytic bacteria are commonly reported to decrease (Fernando et al., 2010; Petri et al., 2013) due to their pH sensitivity (Russell and Dombrowski, 1980). However, the family *Ruminococcacceae* also contains members, which are able to digest starch (Stewart et al., 1997) and, therefore, are sometimes reported to increase during high concentrate feeding trials (Khafipour et al., 2009b; Mao et al., 2013). Members of the ruminal *Spirochaetaceae* are not able to digest cellulose; however, they closely interact with particle adherent cellulolytic bacteria, which provide soluble sugars and carbohydrates (Stanton and Canale-Parola, 1980). Presumably, the SARA period indirectly led to a decrease in *Spirochaetes* when low pH values reduced the abundance of cellulolytic bacteria. The phylum *Lentisphaerae* is reported to be more abundant when high forage feed is supplied (Pitta et al., 2014) and to decrease during times of low pH when SARA was induced in cattle (Mao et al., 2013). However, the role of this bacterial group in ruminal fermentation still remains to be determined (Myer et al., 2015). The minor phylum *Verrucomicrobia* was more abundant in goats exposed to a SARA challenge (Huo et al., 2014) as well as in cattle during a high grain diet in the study by Hook et al. (2011). Results of both studies are contrary to our observations, in which this phylum decreased in both phases during the SARA challenge. The impact of SARA on the abundance of *Tenericutes* also varies among studies; however, a decrease has been described previously in an *in vivo* study in which an enhanced ratio of ground wheat and barley was fed to cattle (Plaizier et al., 2017). As the anaerobic rumen bacteria are difficult to cultivate, information on the fermentation properties of minor groups and reference sequences from these groups for taxonomic classification are still incomplete. Whether these discrepancies are related to differences on lower taxonomic levels might be revealed in the future with the increasing availability of species reference sequences and proteomes.

When physiological pH values were reinduced, most bacterial alterations vanished, and both sequencing approaches revealed a recovery of the bacterial community. However, the phylum *Elusimicrobia* and the families *Bacteroidales BS11 gut group* and *Endomicrobiaceae*, the genera *CPla-4 termite group* and *Prevotellaceae UCG-001*, and a few ASVs did not recover from SARA. The phylum *Elusimicrobia* also decreased in relative abundance during induced SARA *in vivo* (Neubauer et al., 2019); however, it was also diminished in a previous RUSITEC experiment without SARA (Wetzels et al., 2018). Therefore, this change might not be specific for the SARA challenge. The mentioned groups were merely present at very low levels during the whole experiment. In total, richness and diversity indices as well as β-diversity analysis revealed a regeneration process in all treatment groups, proving the RUSITEC to be an adequate *in vitro* alternative to *in vivo* experiments on the recovery from SARA challenges. Despite the closed nature of the RUSITEC system and the frequently discussed limitations especially with regard to protozoa survival (Moumen et al., 2009), the bacterial community members appear to be able to survive challenges in the *in vitro* system and to reestablish the previous community composition. Quantitative PCR rarely revealed a decrease in 16S rRNA gene copy numbers during SARA and no differences among control periods supporting our observations on the high stability of the bacterial community in the *in vitro* system.

The combination of two different sequencing technologies was applied to benefit from both and, furthermore, to validate the results of both data sets against each other. Illumina MiSeq sequencing performed better in covering the almost complete bacterial diversity of the pooled samples and was therefore superior to calculate alpha and beta diversity metrics. The longer reads generated by PacBio amplicon sequencing were used to gain detailed insights into the taxonomic distribution of the highly abundant phylotypes at the ASV level as well as the composition of the bacterial microbiota at higher taxonomic levels. However, not only are longer read lengths needed for a higher taxonomic resolution, but also the presence of respective best hits in the reference databases. In the Illumina data set, only 0.3% of all ASVs could be assigned to a species, and 0.9% of all PacBio ASVs could be classified at species level. However, due to a lack of best hits in the reference database, the taxonomic resolution in the PacBio data set was not as high as expected. Differences between the Illumina MiSeq and PacBio sequencing results can, furthermore, be explained by the different sequencing depths between the two methods. However, the most abundant phylotypes detected with the PacBio sequencing method were confirmed to be highly abundant in the Illumina data set, indicating that a combination of both sequencing techniques can improve data validation.

In this study, we were able to observe the influence of SARA on the bacterial community in an *in vitro* model. The SARA period had a major impact on the primarily fibrolytic associated bacterial groups, which were diminished during this low pH period. Furthermore, we were able to detect an increase in lactate-producing and -utilizing bacteria. Most observed alterations were visible at several phylogenetic levels. By the end of the experiment, the majority of the bacterial community had recovered from the SARA challenge and equaled the initial abundance. In conclusion, this study implies that the ruminal population is affected by the pH in the RUSITEC model. Some changes were also induced by using a high-concentrate diet. Moreover, this study proved that the bacterial community in the RUSITEC model is able to recover from SARA bouts. Therefore, this *in vitro* model can contribute to enhance the current knowledge of SARA, which can play a major role in several health issues, such as ruminitis, liver abscesses, and more (Gozho et al., 2005; Rezac et al., 2014a, b) and may be used in further studies.

## Data Availability Statement

The datasets generated for this study can be found in the European Nucleotide Archive (ENA) database with accession number PRJEB33637.

## Ethics Statement

The animal study was reviewed and approved by The Lower Saxony State Office for Consumer Protection and Food Safety (LAVES, Post box 3949 in 26029 Oldenburg) approved the previous fistulation of the donor cows by the experiment number AZ 33.4-42505-04-13A373.

## Author Contributions

GB, SW, and MB contributed to the conception and design of the study. TO, MB, and MD performed all lab work and data acquisition. MB, TO, MD, BZ, and SW analyzed and interpreted the data. BP, F-FR, and MB performed the statistical analysis. MB and TO wrote the first draft of the manuscript. MB, MD, and SW wrote sections of the manuscript. All authors contributed to manuscript revision, read and approved the submitted version.

## Conflict of Interest

The authors declare that the research was conducted in the absence of any commercial or financial relationships that could be construed as a potential conflict of interest.

## Footnotes

1 https://www.pacb.com/wp-content/uploads/Procedure-Checklist-%E2%80%93-Amplification-of-Full-Length-16S-Gene-with-Barcoded-Primers-for-Multiplexed-SMRTbell-Library-Preparation-and-Sequencing.pdf

2 https://www.pacb.com/wp-content/uploads/Procedure-Checklist-Full-Length-16S-Amplification-SMRTbell-Library-Preparation-and-Sequencing.pdf

3 https://www.pacb.com/products-and-services/analytical-software/smrt-analysis/

4 https://www.viennabiocenter.org

5 http://www.microbesonline.org/fasttree/
