# Supplementary material for: PacBio and Illumina MiSeq Amplicon Sequencing Confirm Full Recovery of the Bacterial Community After Subacute Ruminal Acidosis Challenge in the RUSITEC System
Source: Front Microbiol. 2020 Aug 7;11:1813. doi: 10.3389/fmicb.2020.01813 (PMC7426372; doi:10.3389/fmicb.2020.01813)
Supplement: Supplementary file 1 [file Data_Sheet_1.PDF]

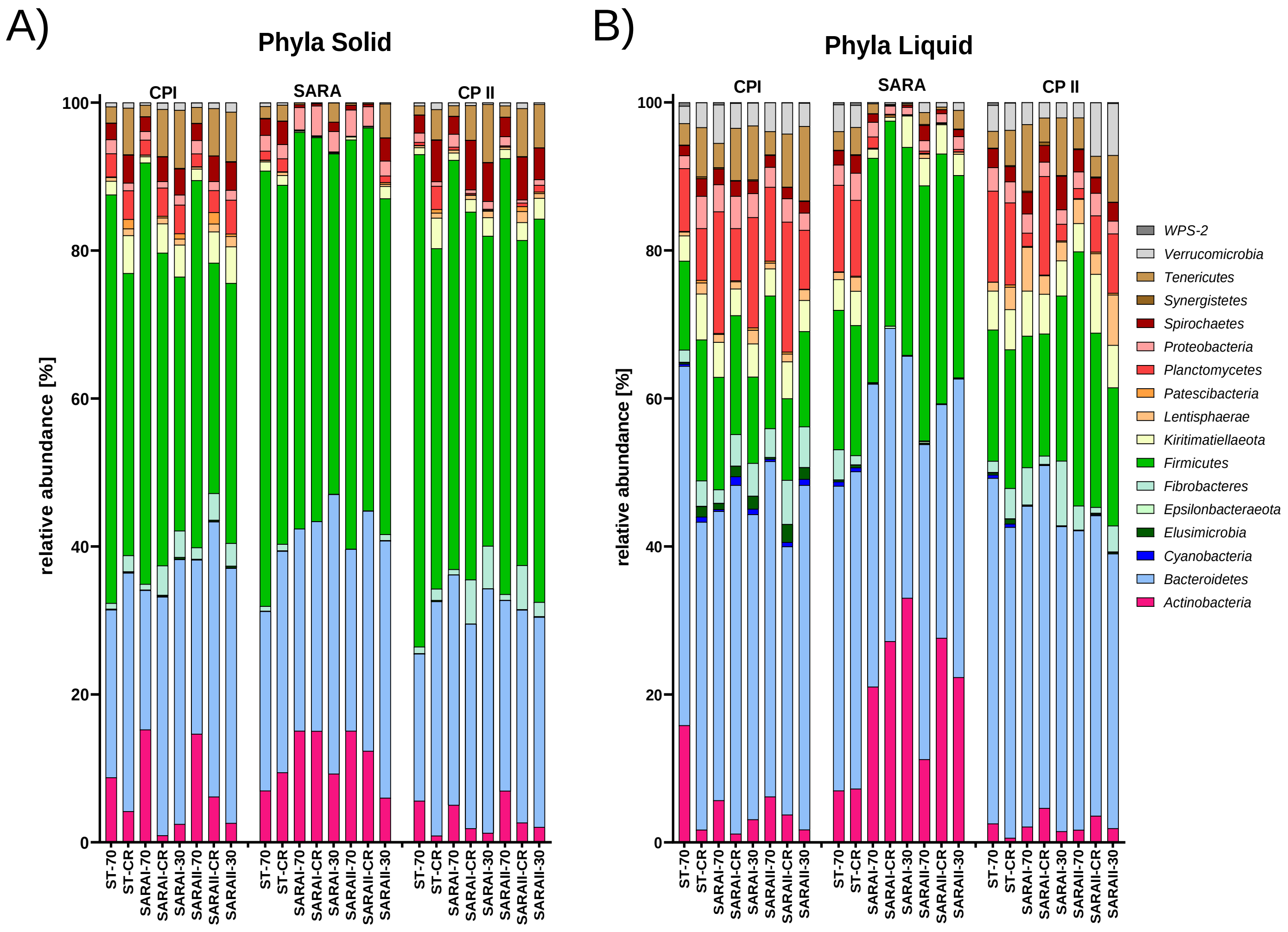

Supplementary Figure 1: Relative abundances on phylum level as detected by the PacBio amplicon sequencing approach for the solid (A) and liquid (B) phase. Relative abundances for each treatment group are presented for all three experimental periods (CP I = control period I, SARA = subacute acidosis period, CP II = control period II). Treatment groups are labeled as following: ST-70 = standard buffer, 70% concentrate; ST-CR = standard buffer, changing ratio; SARAI-70 = SARA I buffer, 70% concentrate; SARAI-30 = SARA I buffer, 30% concentrate; SARAI-CR = SARA I buffer, changing ratio; SARAII-70 = SARA II buffer, 70% concentrate; SARAII-30 = SARA II buffer, 30% concentrate; SARAII-CR = SARA II buffer, changing ratio.
