# Supplementary material for: PacBio and Illumina MiSeq Amplicon Sequencing Confirm Full Recovery of the Bacterial Community After Subacute Ruminal Acidosis Challenge in the RUSITEC System
Source: Front Microbiol. 2020 Aug 7;11:1813. doi: 10.3389/fmicb.2020.01813 (PMC7426372; doi:10.3389/fmicb.2020.01813)
Supplement: Supplementary file 2 [file Data_Sheet_2.PDF]

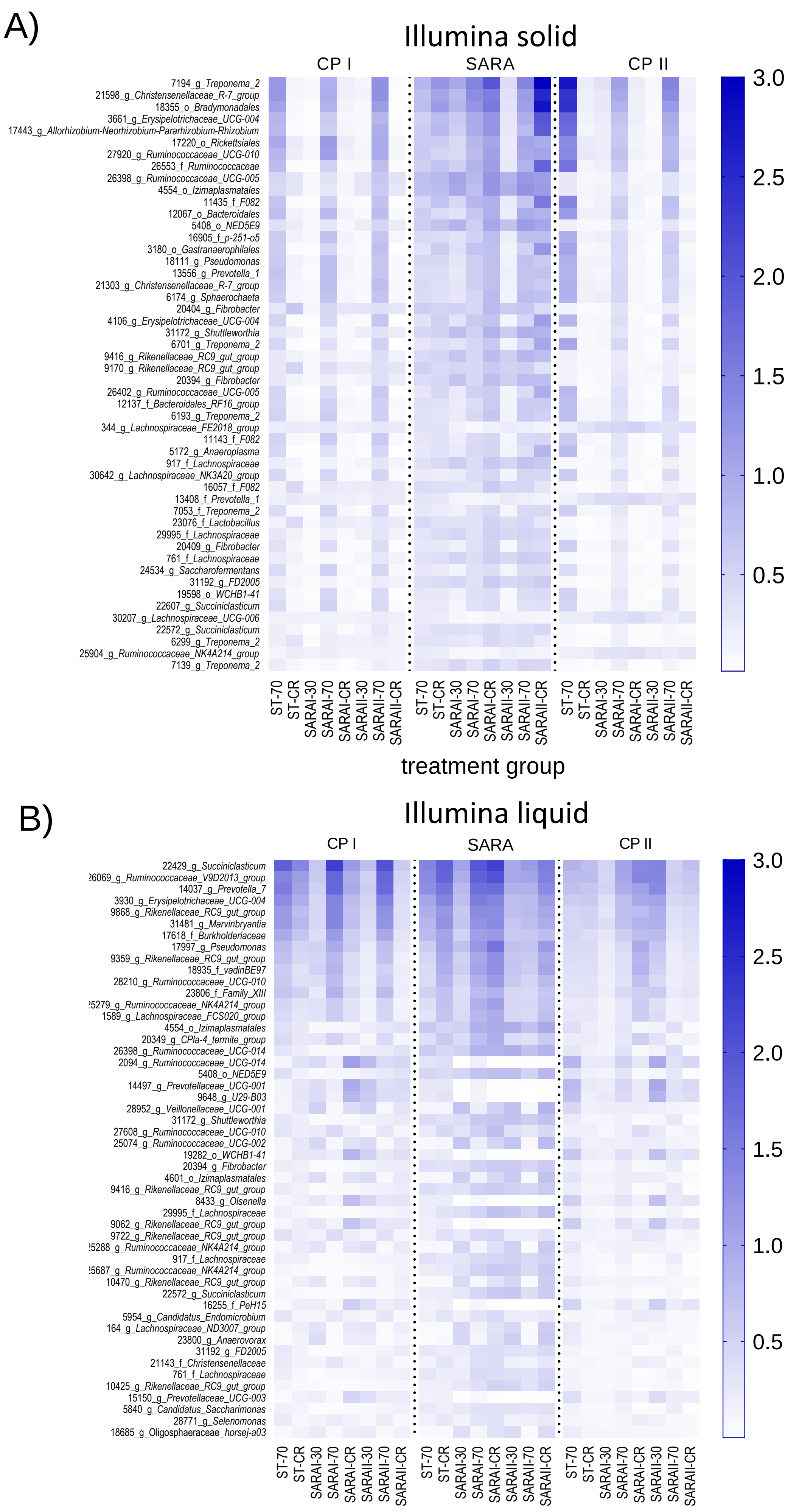

Supplementary Figure 2

Supplementary figure 2: Alterations of the 50 most abundant amplicon sequence variants (ASVs) in the Illumina analysis throughout all experimental periods (left: CP I = control period I, middle: AP = acidosis period, right: CP II = control period II) for the solid (A) and liquid (B) phase. ASVs are labeled with the ASV-number, followed by the lowest classification level (o = order, f = family, g = genus) and the taxonomic name. Treatment groups were pooled from all four runs and labeled as following: ST-70 = standard buffer, 70% concentrate; ST-CR = standard buffer, changing ratio; SARAI-70 = SARA I buffer, 70% concentrate; SARAI-30 = SARA I buffer, 30% concentrate; SARAI-CR = SARA I buffer, changing ratio; SARAII-70 = SARA I buffer, 70% concentrate; SARAI-30 = SARA I buffer, 30% concentrate; SARAI-CR = SARA I buffer, changing ratio. The relative abundance [%] is indicated by the color scale.
