# Supplementary material for: PacBio and Illumina MiSeq Amplicon Sequencing Confirm Full Recovery of the Bacterial Community After Subacute Ruminal Acidosis Challenge in the RUSITEC System
Source: Front Microbiol. 2020 Aug 7;11:1813. doi: 10.3389/fmicb.2020.01813 (PMC7426372; doi:10.3389/fmicb.2020.01813)
Supplement: Supplementary file 3 [file Data_Sheet_3.PDF]

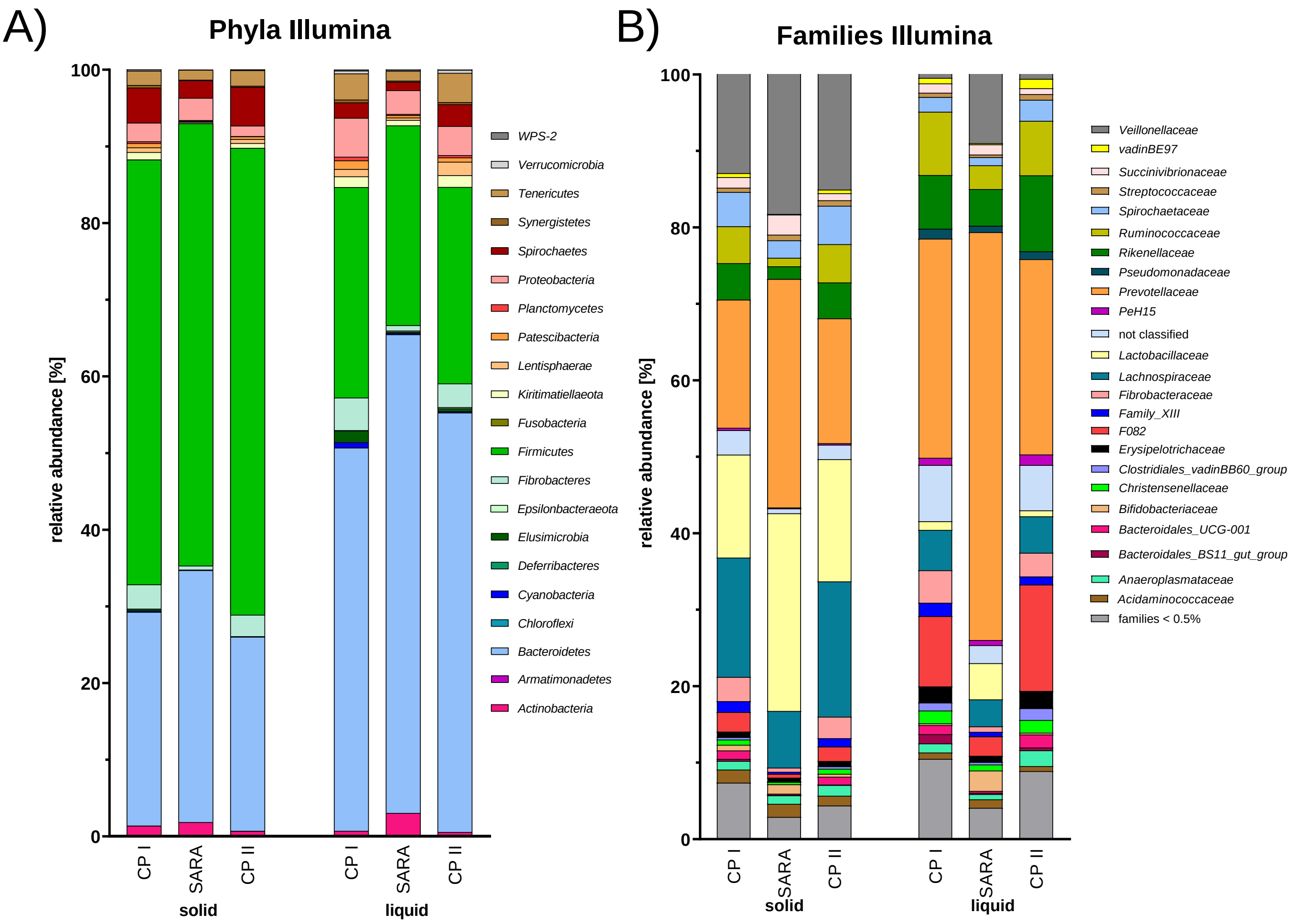

Supplementary Figure 3: Relative abundances of bacterial phyla (A) and families (B) in solid and liquid phase samples as measured by Illumina MiSeq amplicon sequencing of the V4 region of the 16S rRNA gene. Families with an abundance of less than 0.5% were combined into one group. Pooled samples from all four runs were used per treatment group at each time-point (CP I = control period I, SARA = subacute acidosis period, CP II = control period II). Treatments were labeled as following: ST-70 = standard buffer, 70% concentrate; ST-CR = standard buffer, changing ratio; SARAI-70 = SARA I buffer, 70% concentrate; SARAI-30 = SARA I buffer, 30% concentrate; SARAI-CR = SARA I buffer, changing ratio; SARAII-70 = SARA II buffer, 70% concentrate; SARAII-30 = SARA II buffer, 30% concentrate; SARAII-CR = SARA II buffer, changing ratio.
