# Supplementary material for: PacBio and Illumina MiSeq Amplicon Sequencing Confirm Full Recovery of the Bacterial Community After Subacute Ruminal Acidosis Challenge in the RUSITEC System
Source: Front Microbiol. 2020 Aug 7;11:1813. doi: 10.3389/fmicb.2020.01813 (PMC7426372; doi:10.3389/fmicb.2020.01813)
Supplement: Supplementary file 6 [file Data_Sheet_6.PDF]

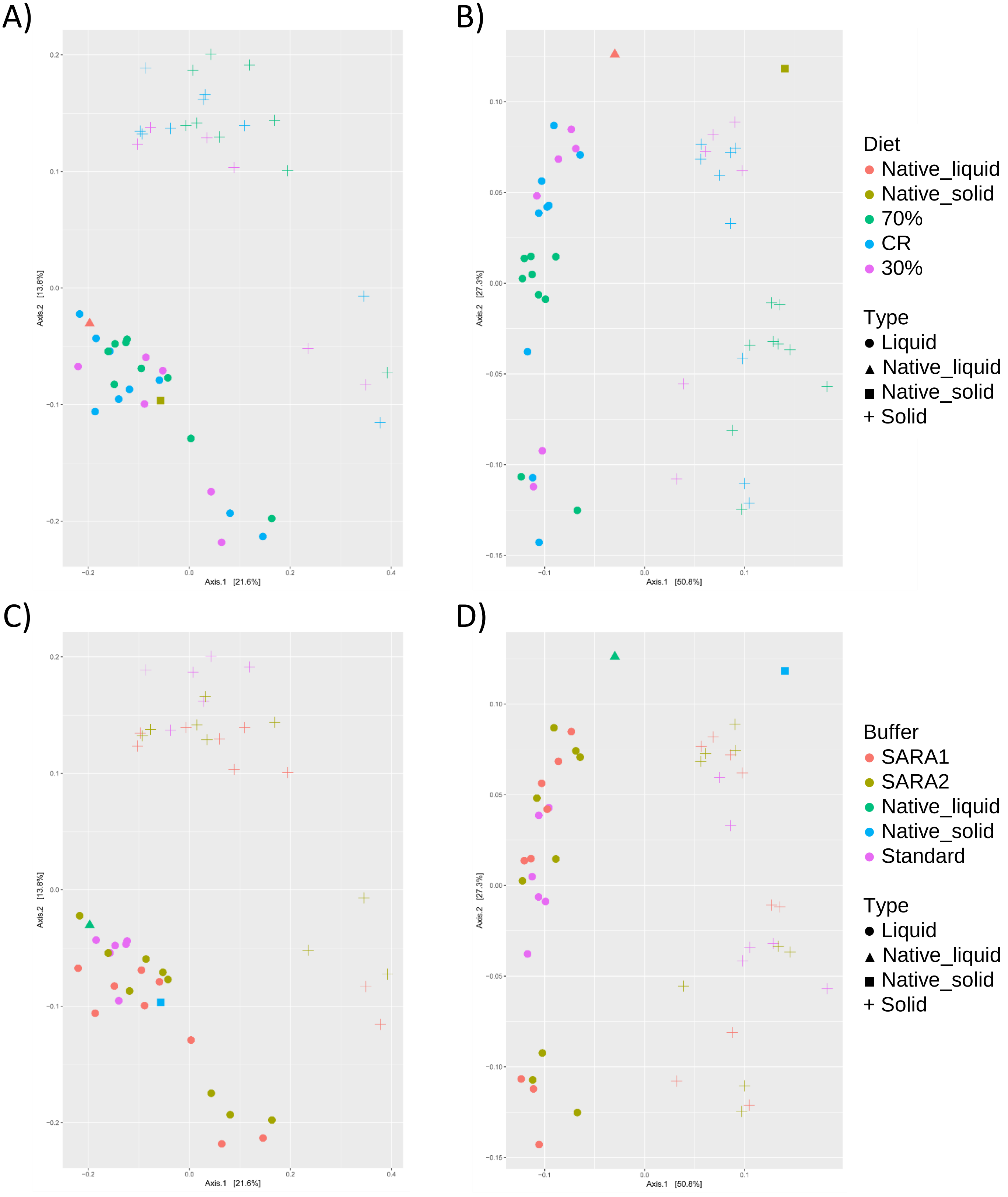

Supplementary Figure 6: Unweighted (A) and weighted (B) UniFrac distances among samples labeled according to the diet and unweighted (C) and weighted (D) UniFrac distances labeled according to the buffer type.
