# Supplementary material for: PacBio and Illumina MiSeq Amplicon Sequencing Confirm Full Recovery of the Bacterial Community After Subacute Ruminal Acidosis Challenge in the RUSITEC System
Source: Front Microbiol. 2020 Aug 7;11:1813. doi: 10.3389/fmicb.2020.01813 (PMC7426372; doi:10.3389/fmicb.2020.01813)
Supplement: Supplementary file 7 [file Data_Sheet_7.PDF]

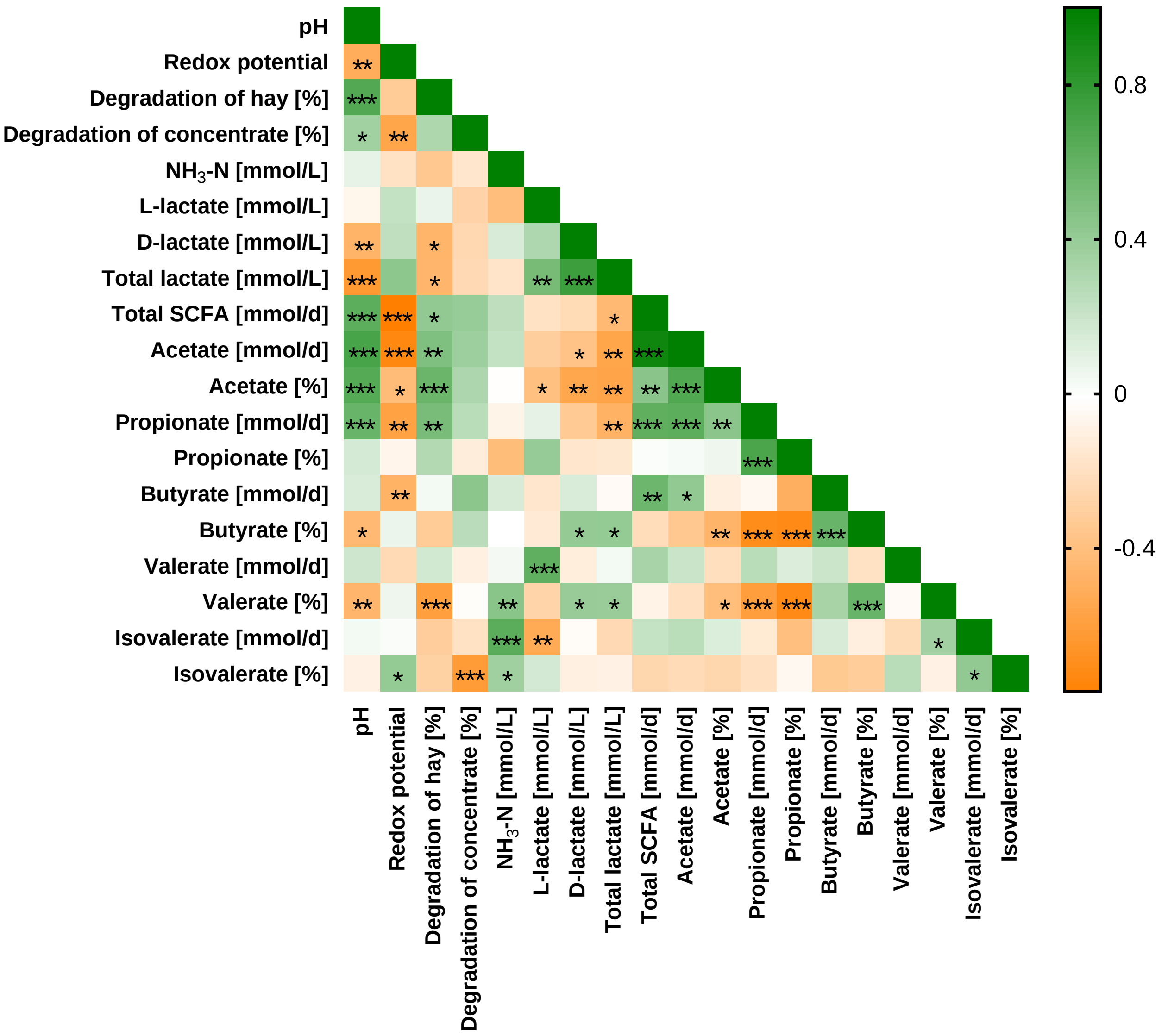

Supplementary figure 7: Spearman correlations among biochemical parameters on day 17 of this experiment. Spearman ρ is indicated by the green to orange color scheme. Significant correlations are indicated by * $P < 0.05$, ** $P < 0.01$, *** $P < 0.001$.
