# Supplementary material for: PacBio and Illumina MiSeq Amplicon Sequencing Confirm Full Recovery of the Bacterial Community After Subacute Ruminal Acidosis Challenge in the RUSITEC System
Source: Front Microbiol. 2020 Aug 7;11:1813. doi: 10.3389/fmicb.2020.01813 (PMC7426372; doi:10.3389/fmicb.2020.01813)
Supplement: Supplementary file 8 [file Table_1.DOCX]

**Supplementary Table 1**.

|  | Type of buffer | | |
| --- | --- | --- | --- |
| Substance [mmol/l] | Standard | SARAI | SARAII |
| NaCl | 28 | 115.9 | 110.9 |
| KCl | 7.69 | 7.69 | 7.69 |
| CaCl_2_ ∙ 2 H_2_O | 0.216 | 0.216 | 0.216 |
| MgCl_2_ ∙ 6 H_2_O | 0.63 | 0.63 | 0.63 |
| HCl (1 N) | 0.5 | 0.5 | 0.5 |
| NaH_2_PO_4_ ∙ H_2_O | 10 | 10 | 10 |
| Na_2_HPO_4_ ∙ 12 H_2_O | 10 | - | - |
| NH_4_Cl | 5 | 5 | 5 |
| NaHCO_3_ | 97.9 | 20 | 25 |

The pH value within the RUSITEC vessels were influenced by constantly infusing a buffer solution. The physiological pH was maintained by applying the standard buffer. During subacute rumen acidosis (SARA) period, vessels of all treatment groups, were infused with SARAI or SARAII buffer solutions to induce SARA conditions.
