# Supplementary material for: PacBio and Illumina MiSeq Amplicon Sequencing Confirm Full Recovery of the Bacterial Community After Subacute Ruminal Acidosis Challenge in the RUSITEC System
Source: Front Microbiol. 2020 Aug 7;11:1813. doi: 10.3389/fmicb.2020.01813 (PMC7426372; doi:10.3389/fmicb.2020.01813)
Supplement: Supplementary file 9 [file Table_2.DOCX]

**Supplementary Table 2.** The 50 most abundant genera in both sequencing approaches with their mean relative abundance in all samples.

| Illumina | | PacBio | |
| --- | --- | --- | --- |
| Genus | Abun-dance  [%] | Genus | Abun-dance  [%] |
| Rikenellaceae_RC9_gut_group | 6.94 | Lactobacillus | 13.96 |
| Treponema_2 | 6.56 | Prevotella_1 | 13.82 |
| Christensenellaceae_R-7_group | 3.79 | Rikenellaceae_RC9_gut_group | 8.08 |
| Ruminococcaceae_UCG-010 | 3.09 | Bifidobacterium | 7.82 |
| Ruminococcaceae_NK4A214_group | 3.08 | CPla-4_termite_group | 3.48 |
| Prevotella_1 | 3.04 | Fibrobacter | 2.32 |
| Erysipelotrichaceae_UCG-004 | 2.97 | Prevotella_7 | 2.26 |
| Succiniclasticum | 2.56 | Selenomonas | 2.08 |
| Fibrobacter | 2.50 | Treponema_2 | 1.93 |
| Anaeroplasma | 1.70 | Selenomonas_1 | 1.89 |
| Prevotellaceae_UCG-003 | 1.57 | Prevotellaceae_YAB2003_group | 1.46 |
| Selenomonas_1 | 1.40 | Prevotellaceae_UCG-001 | 1.25 |
| Butyrivibrio_2 | 1.36 | Butyrivibrio_2 | 1.13 |
| Ruminococcaceae_UCG-014 | 1.24 | Saccharofermentans | 1.11 |
| Pseudomonas | 1.06 | Schwartzia | 1.07 |
| Anaerovorax | 1.05 | Oribacterium | 1.02 |
| Ruminococcaceae_UCG-002 | 1.02 | Megasphaera | 0.97 |
| Veillonellaceae_UCG-001 | 0.93 | Anaerovibrio | 0.90 |
| Ruminococcaceae_UCG-005 | 0.92 | Pseudobutyrivibrio | 0.76 |
| Prevotellaceae_UCG-001 | 0.91 | Anaerobiospirillum | 0.74 |
| Candidatus_Saccharimonas | 0.90 | Streptococcus | 0.71 |
| Prevotella_7 | 0.84 | Anaeroplasma | 0.61 |
| Lachnospiraceae_NK3A20_group | 0.79 | p-1088-a5_gut_group | 0.59 |
| Lachnospiraceae_FCS020_group | 0.73 | Lachnospiraceae_AC2044_group | 0.55 |
| CPla-4_termite_group | 0.73 | Prevotellaceae_UCG-003 | 0.53 |
| Ruminococcaceae_V9D2013_group | 0.72 | Erysipelotrichaceae_UCG-004 | 0.53 |
| Lactobacillus | 0.68 | Veillonellaceae_UCG-001 | 0.51 |
| Papillibacter | 0.63 | Sphaerochaeta | 0.44 |
| Acetitomaculum | 0.62 | horsej-a03 | 0.43 |
| Oribacterium | 0.62 | Dialister | 0.37 |
| Olsenella | 0.58 | Succiniclasticum | 0.34 |
| Prevotellaceae_YAB2003_group | 0.55 | Pseudomonas | 0.30 |
| Marvinbryantia | 0.54 | Lachnoclostridium_1 | 0.29 |
| Lachnospiraceae_NK4A136_group | 0.51 | Lachnospiraceae_NK3A20_  group | 0.29 |
| Allorhizobium-Neorhizobium-Pararhizobium-Rhizobium | 0.48 | Ruminococcus_1 | 0.27 |
| Anaerovibrio | 0.47 | Syntrophococcus | 0.27 |
| FD2005 | 0.44 | Olsenella | 0.26 |
| Candidatus_Endomicrobium | 0.44 | Anaerovorax | 0.26 |
| Shuttleworthia | 0.43 | Ruminococcaceae_UCG-010 | 0.26 |
| Ruminococcus_1 | 0.43 | Candidatus_Endomicrobium | 0.24 |
| horsej-a03 | 0.39 | Acetitomaculum | 0.23 |
| U29-B03 | 0.38 | Family_XIII_AD3011_group | 0.22 |
| Lachnospiraceae_ND3007_group | 0.38 | probable_genus_10 | 0.21 |
| Lachnoclostridium_10 | 0.35 | Christensenellaceae_R-7_group | 0.21 |
| Ruminococcaceae_UCG-004 | 0.34 | Roseburia | 0.21 |
| Selenomonas | 0.32 | Acidaminococcus | 0.20 |
| Sphaerochaeta | 0.32 | Desulfovibrio | 0.20 |
| Saccharofermentans | 0.32 | Candidatus_Saccharimonas | 0.19 |
| Lachnospiraceae_FE2018_group | 0.32 | Lachnospiraceae_NK4A136_  group | 0.18 |
| Ruminococcaceae_UCG-013 | 0.31 | Succinivibrio | 0.17 |
