# Supplementary material for: PacBio and Illumina MiSeq Amplicon Sequencing Confirm Full Recovery of the Bacterial Community After Subacute Ruminal Acidosis Challenge in the RUSITEC System
Source: Front Microbiol. 2020 Aug 7;11:1813. doi: 10.3389/fmicb.2020.01813 (PMC7426372; doi:10.3389/fmicb.2020.01813)
Supplement: Supplementary file 10 [file Table_3.DOCX]

**Supplementary Table 3. Significant changes on phylum level detected by PacBio sequencing.**

|  |  | solid phase | | |  | liquid phase | | |
| --- | --- | --- | --- | --- | --- | --- | --- | --- |
|  |  |  | period^1^ |  |  |  | period^1^ |  |
|  |  | **CP I –**  **SARA** | **SARA –**  **CP II** | **CP I – CP II** |  | **CP I –**  **SARA** | **SARA – CP II** | **CP I –**  **CP II** |
| phylum^2^ | treatment group^3^ | *P*-value^4^ | *P*-value^4^ | *P*-value^4^ |  | *P*-value^4^ | *P*-value^4^ | *P*-value^4^ |
| *Elusimicrobia* | SARAI-70 | n.s. | n.s. | n.s. |  | 0.023 | n.s. | n.s. |
|  | SARAI-30 | n.s. | n.s. | n.s. |  | n.s. | n.s. | 0.003 |
|  | SARAI-CR | 0.024 | n.s. | 0.007 |  | 0.024 | n.s. | 0.007 |
|  | SARAII-30 | 0.005 | n.s. | n.s. |  | n.s. | n.s. | 0.016 |
|  | SARAII-CR | n.s. | n.s. | n.s. |  | 0.008 | n.s. | n.s. |
| *Fibrobacteres* | SARAI-70 | 0.014 | n.s. | n.s. |  | 0.019 | n.s. | n.s. |
|  | SARAI-30 | n.s. | 0.008 | n.s. |  | 0.019 | n.s. | n.s. |
|  | SARAI-CR | n.s. | 0.019 | n.s. |  | 0.019 | n.s. | n.s. |
|  | SARAII-70 | 0.011 | n.s. | n.s. |  | 0.006 | n.s. | n.s. |
|  | SARAII-30 | n.s. | n.s. | n.s. |  | 0.015 | n.s. | n.s. |
|  | SARAII-CR | n.s. | 0.009 | n.s. |  | 0.014 | n.s. | n.s. |
| *Kiritimatiellaeota* | SARAI-70 | n.s. | n.s. | n.s. |  | n.s. | 0.021 | n.s. |
|  | SARAI-30 | 0.003 | n.s. | n.s. |  | 0.016 | n.s. | n.s. |
|  | SARAI-CR | 0.003 | n.s. | n.s. |  | n.s. | n.s. | n.s. |
|  | SARAII-30 | 0.004 | n.s. | n.s. |  | n.s. | n.s. | n.s. |
|  | SARAII-CR | 0.005 | n.s. | n.s. |  | n.s. | n.s. | n.s. |
|  | ST-CR | 0.021 | n.s. | n.s. |  | n.s. | n.s. | n.s. |
| *Lentisphaerae* | SARAI-70 | n.s. | n.s. | n.s. |  | n.s. | 0.011 | n.s. |
|  | SARAI-30 | n.s. | 0.011 | n.s. |  | n.s. | 0.003 | n.s. |
|  | SARAI-CR | 0.0115 | n.s. | n.s. |  | n.s. | 0.003 | n.s. |
|  | SARAII-70 | n.s. | n.s. | n.s. |  | n.s. | 0.005 | n.s. |
|  | SARAII-30 | 0.012 | n.s. | n.s. |  | n.s. | 0.008 | n.s. |
|  | SARAII-CR | n.s. | 0.020 | n.s. |  | n.s. | 0.007 | n.s. |
| *Planctomycetes* | SARAI-70 | n.s. | n.s. | n.s. |  | 0.015 | n.s. | n.s. |
|  | SARAI-30 | 0.021 | n.s. | n.s. |  | n.s. | n.s. | n.s. |
|  | SARAI-CR | 0.007 | n.s. | n.s. |  | 0.014 | n.s. | n.s. |
|  | SARAII-70 | 0.014 | n.s. | n.s. |  | n.s. | n.s. | n.s. |
|  | SARAII-30 | 0.014 | n.s. | n.s. |  | 0.015 | n.s. | n.s. |
|  | SARAII-CR | 0.009 | n.s. | n.s. |  | 0.009 | n.s. | n.s. |
| *Spirochaetes* | SARAI-70 | n.s. | 0.012 | n.s. |  | n.s. | n.s. | n.s. |
|  | SARAI-30 | n.s. | 0.003 | n.s. |  | n.s. | n.s. | n.s. |
|  | SARAI-CR | n.s. | 0.004 | n.s. |  | n.s. | 0.005 | n.s. |
|  | SARAII-CR | n.s. | 0.007 | n.s. |  | n.s. | n.s. | n.s. |
| *Tenericutes* | SARAI-70 | n.s. | n.s. | n.s. |  | n.s. | 0.016 | n.s. |
|  | SARAI-30 | n.s. | 0.021 | n.s. |  | n.s. | 0.016 | n.s. |
|  | SARAI-CR | 0.012 | n.s. | n.s. |  | n.s. | 0.016 | n.s. |
|  | SARAII-70 | 0.021 | n.s. | n.s. |  | 0.021 | n.s. | n.s. |
|  | SARAII-CR | 0.016 | n.s. | n.s. |  | n.s. | n.s. | n.s. |
|  | ST-CR | 0.005 | n.s. | n.s. |  | n.s. | n.s. | n.s. |
| *Verrucomicrobia* | SARAI-70 | n.s. | 0.021 | n.s. |  | 0.007 | n.s. | n.s. |
|  | SARAI-30 | n.s. | 0.003 | n.s. |  | 0.021 | n.s. | n.s. |
|  | SARAI-CR | n.s. | 0.009 | n.s. |  | 0.012 | n.s. | n.s. |
|  | SARAII-30 | 0.005 | n.s. | n.s. |  | n.s. | n.s. | n.s. |

^1^period: CP I = control period I; SARA = subacute rumen acidosis period; CP II = control period II

^2^ only phyla with significant changes are listed

^3^Treatment groups: SARAI-70 = SARA I buffer, 70% concentrate; SARAI-30 = SARA I buffer, 30%; SARAI-CR = SARA I buffer, changing ratio; SARAII-70 = SARA II buffer, 70% concentrate; SARAII-30 = SARA II buffer, 30%; SARAII-CR = SARA II buffer, changing ratio; ST-CR = Standard buffer, changing ratio. Only groups with significant changes are shown for each phylum.

^4^ n.s. = not significant
