# Supplementary material for: PacBio and Illumina MiSeq Amplicon Sequencing Confirm Full Recovery of the Bacterial Community After Subacute Ruminal Acidosis Challenge in the RUSITEC System
Source: Front Microbiol. 2020 Aug 7;11:1813. doi: 10.3389/fmicb.2020.01813 (PMC7426372; doi:10.3389/fmicb.2020.01813)
Supplement: Supplementary file 11 [file Table_4.DOCX]

**Supplementary Table 4. Significant changes on family level detected using PacBio sequencing for families with a total relative abundance of > 0.5 %**

|  |  | solid phase | | |  | liquid phase | | |
| --- | --- | --- | --- | --- | --- | --- | --- | --- |
|  |  | period^1^ | | |  | period^1^ | | |
| family^2^ | treatment group^3^ | **CP I - SARA** | **SARA**  **-CP II** | **CP I -  CP II** |  | **CP I - SARA** | **SARA -CP II** | **CP I -  CP II** |
|  |  | *P*-value^4^ | *P*-value^4^ | *P*-value^4^ |  | *P*-value^4^ | *P*-value^4^ | *P*-value^4^ |
| *Bacteroidales_*  *BS11_gut_group* | SARAI-70 | 0.015 | n.s. | 0.021 |  | n.s. | n.s. | n.s. |
|  | SARAI-30 | n.s. | n.s. | 0.009 |  | n.s. | n.s. | n.s. |
|  | SARAI-CR | n.s. | n.s. | n.s. |  | 0.016 | n.s. | n.s. |
|  | SARAII-70 | 0.015 | n.s. | n.s. |  | n.s. | n.s. | n.s. |
|  | SARAII-30 | n.s. | n.s. | 0.009 |  | n.s. | n.s. | n.s. |
| *Bacteroidales_ UCG-001* | SARAI-70 | n.s. | 0.019 | n.s. |  | n.s. | 0.011 | n.s. |
|  | SARAI-30 | n.s. | 0.019 | n.s. |  | n.s. | 0.008 | n.s. |
|  | SARAI-CR | 0.025 | 0.025 | n.s. |  | n.s. | 0.012 | n.s. |
|  | SARAII-70 | 0.007 | n.s. | n.s. |  | 0.015 | 0.015 | n.s. |
|  | SARAII-30 | n.s. | 0.019 | n.s. |  | n.s. | n.s. | n.s. |
|  | SARAII-CR | n.s. | 0.019 | n.s. |  | n.s. | 0.011 | n.s. |
| *Fibrobacteraceae* | SARAI-70 | 0.014 | n.s. | n.s. |  | 0.019 | n.s. | n.s. |
|  | SARAI-30 | n.s. | 0.008 | n.s. |  | 0.019 | n.s. | n.s. |
|  | SARAI-CR | n.s. | 0.019 | n.s. |  | 0.019 | n.s. | n.s. |
|  | SARAII-70 | 0.011 | n.s. | n.s. |  | 0.006 | n.s. | n.s. |
|  | SARAII-30 | n.s. | 0.008 | n.s. |  | 0.015 | n.s. | n.s. |
|  | SARAII-CR | n.s. | 0.009 | n.s. |  | 0.014 | n.s. | n.s. |
| *Lachnospiraceae* | SARAI-70 | n.s. | 0.016 | n.s. |  | n.s. | n.s. | n.s. |
|  | SARAI-CR | n.s. | 0.007 | n.s. |  | n.s. | n.s. | n.s. |
|  | SARAII-30 | 0.021 | n.s. | n.s. |  | n.s. | n.s. | n.s. |
|  | SARAII-CR | n.s. | 0.009 | n.s. |  | n.s. | n.s. | n.s. |
|  | ST-CR | n.s. | 0.016 | n.s. |  | n.s. | n.s. | n.s. |
| *Lactobacillaceae* | SARAI-30 | n.s. | n.s. | n.s. |  | 0.022 | n.s. | n.s. |
|  | SARAI-CR | n.s. | 0.021 | n.s. |  | n.s. | 0.021 | n.s. |
|  | SARAII-CR | 0.009 | n.s. | n.s. |  | n.s. | 0.007 | n.s. |
|  | ST-CR | 0.021 | n.s. | n.s. |  | n.s. | 0.021 | n.s. |
| *Pedosphaeraceae* | SARAI-70 | n.s. | n.s. | n.s. |  | 0.007 | n.s. | n.s. |
|  | SARAI-30 | 0.003 | n.s. | n.s. |  | 0.021 | n.s. | n.s. |
|  | SARAI-CR | 0.009 | n.s. | n.s. |  | 0.012 | n.s. | n.s. |
|  | SARAII-30 | 0.003 | n.s. | n.s. |  | n.s. | n.s. | n.s. |
| *Pirellulaceae* | SARAI-70 | n.s. | n.s. | n.s. |  | 0.015 | n.s. | n.s. |
|  | SARAI-30 | 0.021 | n.s. | n.s. |  | n.s. | n.s. | n.s. |
|  | SARAI-CR | 0.007 | n.s. | n.s. |  | 0.019 | n.s. | n.s. |
|  | SARAII-70 | 0.014 | n.s. | n.s. |  | n.s. | n.s. | n.s. |
|  | SARAII-30 | 0.021 | n.s. | n.s. |  | 0.015 | n.s. | n.s. |
|  | SARAII-CR | 0.016 | n.s. | n.s. |  | 0.009 | n.s. | n.s. |
| *Prevotellaceae* | SARAI-70 | n.s. | n.s. | n.s. |  | n.s. | 0.011 | n.s. |
|  | SARAI-30 | n.s. | 0.016 | n.s. |  | n.s. | n.s. | n.s. |
|  | SARAII-30 | n.s. | 0.016 | n.s. |  | n.s. | n.s. | n.s. |
|  | SARAII-CR | n.s. | 0.016 | n.s. |  | n.s. | 0.004 | n.s. |
| *Rikenellaceae* | SARAI-30 | 0.016 | n.s. | n.s. |  | n.s. | n.s. | 0.016 |
|  | SARAI-CR | n.s. | 0.021 | n.s. |  | n.s. | 0.005 | n.s. |
|  | SARAII-70 | 0.021 | n.s. | n.s. |  | n.s. | n.s. | n.s. |
|  | SARAII-30 | 0.016 | n.s. | n.s. |  | n.s. | n.s. | n.s. |
|  | SARAII-CR | n.s. | 0.009 | n.s. |  | n.s. | n.s. | n.s. |
|  | ST-CR | 0.016 | n.s. | n.s. |  | n.s. | 0.016 | n.s. |
| *Ruminococcaceae* | SARAI-70 | n.s. | n.s. | n.s. |  | 0.009 | n.s. | n.s. |
|  | SARAI-30 | n.s. | 0.020 | n.s. |  | n.s. | n.s. | n.s. |
|  | SARAI-CR | n.s. | 0.008 | n.s. |  | n.s. | 0.016 | n.s. |
|  | SARAII-70 | 0.016 | n.s. | n.s. |  | n.s. | n.s. | n.s. |
|  | SARAII-30 | n.s. | 0.020 | n.s. |  | n.s. | n.s. | n.s. |
|  | SARAII-CR | n.s. | 0.011 | n.s. |  | n.s. | n.s. | n.s. |
| *Spirochaetaceae* | SARAI-70 | n.s. | 0.012 | n.s. |  | n.s. | n.s. | n.s. |
|  | SARAI-30 | n.s. | 0.003 | n.s. |  | n.s. | n.s. | n.s. |
|  | SARAI-CR | n.s. | 0.003 | n.s. |  | n.s. | 0.007 | n.s. |
|  | SARAII-30 | n.s. | 0.003 | n.s. |  | n.s. | n.s. | n.s. |
|  | SARAII-CR | n.s. | 0.007 | n.s. |  | n.s. | n.s. | n.s. |
| *Veillonellaceae* | SARAI-30 | n.s. | n.s. | n.s. |  | 0.021 | n.s. | n.s. |
|  | SARAII-30 | n.s. | n.s. | n.s. |  | n.s. | 0.021 | n.s. |
|  | SARAII-CR | n.s. | n.s. | n.s. |  | 0.012 | n.s. | n.s. |

^1^period: CP I = control period I; SARA = SARA period; CP II = control period II

^2^ only families with significant changes are listed

^3^Treatment groups: SARAI-70 = SARA I buffer, 70% concentrate; SARAI-30 = SARA I buffer, 30%; SARAI-CR = SARA I buffer, changing ratio; SARAII-70 = SARA II buffer, 70% concentrate; SARAII-30 = SARA II buffer, 30%; SARAII-CR = SARA II buffer, changing ratio; ST-CR = Standard buffer, changing ratio. Only groups with significant changes are displayed.

^4^ n.s. = not significant
