# Supplementary material for: PacBio and Illumina MiSeq Amplicon Sequencing Confirm Full Recovery of the Bacterial Community After Subacute Ruminal Acidosis Challenge in the RUSITEC System
Source: Front Microbiol. 2020 Aug 7;11:1813. doi: 10.3389/fmicb.2020.01813 (PMC7426372; doi:10.3389/fmicb.2020.01813)
Supplement: Supplementary file 12 [file Table_5.DOCX]

**Supplementary Table 5. Significant changes on genus level detected using PacBio sequencing for the 25 most abundant genera in solid or liquid phase**

|  |  | solid phase | | |  | liquid phase | | |
| --- | --- | --- | --- | --- | --- | --- | --- | --- |
|  |  | period^1^ | | |  | period^1^ | | |
| genus^2^ | treatment group^3^ | **CP I - SARA** | **SARA -CP II** | **CP I -  CP II** |  | **CP I - SARA** | **SARA -CP II** | **CP I -  CP II** |
|  |  | *P*-value^4^ | *P*-value^4^ | *P*-value^4^ |  | *P*-value^4^ | *P*-value^4^ | *P*-value^4^ |
| *Acetitomaculum^5^* | SARAI-CR |  |  |  |  | n.s. | 0.016 | n.s. |
| *Anaeroplasma* | SARAI-70 | n.s. | n.s. | n.s. |  | n.s. | 0.016 | n.s. |
|  | SARAI-CR | 0.016 | n.s. | n.s. |  | n.s. | 0.004 | n.s. |
|  | SARAII-30 | n.s. | n.s. | n.s. |  | n.s. | 0.012 | n.s. |
| *Butyrivibrio 2^5^* | SARAI-CR | n.s. | 0.016 | n.s. |  |  |  |  |
|  | SARAII-70 | 0.021 | n.s. | n.s. |  |  |  |  |
|  | SARAII-30 | n.s. | n.s. | 0.016 |  |  |  |  |
|  | SARAII-CR | n.s. | 0.010 | n.s. |  |  |  |  |
| *CPla-4 termite group* | SARAI-70 | n.s. | n.s. | n.s. |  | 0.015 | n.s. | n.s. |
|  | SARAI-30 | n.s. | n.s. | 0.003 |  | n.s. | n.s. | n.s. |
|  | SARAI-CR | n.s. | n.s. | 0.020 |  | 0.020 | n.s. | n.s. |
|  | SARAII-70 | 0.008 | n.s. | n.s. |  | n.s. | n.s. | n.s. |
|  | SARAII-30 | n.s. | n.s. | 0.019 |  | 0.015 | n.s. | n.s. |
|  | SARAII-CR | 0.020 | n.s. | n.s. |  | 0.021 | n.s. | n.s. |
| *Erysipelotricha-ceae UCG-004^5^* | SARAI-70 |  |  |  |  | n.s. | 0.016 | n.s. |
|  | SARAI-CR |  |  |  |  | n.s. | 0.006 | n.s. |
|  | SARAII-70 |  |  |  |  | n.s. | 0.003 | n.s. |
| *Fibrobacter* | SARAI-70 | 0.014 | n.s. | n.s. |  | n.s. | n.s. | n.s. |
|  | SARAI-30 | n.s. | 0.021 | n.s. |  | n.s. | 0.014 | n.s. |
|  | SARAI-CR | n.s. | 0.019 | n.s. |  | 0.019 | n.s. | n.s. |
|  | SARAII-70 | 0.015 | n.s | n.s |  | 0.006 | n.s | n.s |
|  | SARAII-30 | n.s. | n.s. | n.s. |  | 0.015 | n.s. | n.s. |
|  | SARAII-CR | n.s. | 0.012 | n.s. |  | 0.011 | n.s. | n.s. |
| *horsej-a03^5^* | SARAI-70 |  |  |  |  | n.s. | 0.006 | n.s. |
|  | SARAI-30 |  |  |  |  | n.s. | 0.007 | n.s. |
|  | SARAI-CR |  |  |  |  | n.s. | 0.010 | n.s. |
|  | ST-70 |  |  |  |  | n.s. | n.s. | 0.02 |
|  |  |  |  |  |  |  |  |  |
| *Lachnospiraceae AC2004 group^5^* | SARAI-CR | n.s. | 0.008 | n.s. |  |  |  |  |
|  | SARAII-30 | n.s | 0.014 | n.s |  |  |  |  |
|  | SARAII-CR | n.s | 0.014 | n.s. |  |  |  |  |
| *Lactobacillus* | SARAI-70 | n.s. | n.s. | n.s. |  | 0.010 | n.s. | n.s. |
|  | SARAI-CR | n.s | 0.016 | n.s. |  | n.s. | n.s. | n.s. |
|  | SARAII-CR | 0.012 | n.s. | n.s. |  | 0.007 | n.s. | n.s. |
|  | ST-CR | n.s. | n.s. | n.s. |  | n.s. | 0.021 | n.s. |
| *Oribacterium* | SARAI-30 | n.s. | 0.007 | n.s. |  | n.s. | n.s. | n.s. |
|  | SARAII-30 | n.s. | 0.005 | n.s. |  | n.s. | n.s. | n.s. |
|  | SARAII-CR | 0.012 | n.s. | n.s. |  | n.s. | n.s. | n.s. |
| *p-1088-a5 gut group^5^* | SARAI-70 |  |  |  |  | 0.010 | n.s. | n.s. |
|  | SARAI-30 |  |  |  |  | 0.007 | n.s. | n.s. |
|  | SARAII-30 |  |  |  |  | 0.015 | n.s. | n.s. |
|  | SARAII-CR |  |  |  |  | 0.015 | n.s. | n.s. |
| *Prevotella 1* | SARAII-30 | n.s. | 0.012 | n.s. |  | n.s. | n.s. | n.s. |
|  | ST-CR | n.s. | n.s. | n.s. |  | n.s. | n.s. | 0.020 |
| *Prevotella 7* | SARAI-CR | 0.005 | n.s. | n.s. |  | n.s. | n.s. | n.s. |
|  | SARAII-70 | n.s. | n.s. | 0.007 |  | n.s. | n.s. | n.s. |
|  | SARAII-30 | 0.021 | n.s. | n.s. |  | n.s. | n.s. | n.s. |
| *Prevotellaceae UCG-001* | SARAI-30 | 0.005 | n.s. | n.s. |  | 0.009 | n.s. | n.s. |
|  | SARAI-CR | 0.009 | n.s. | n.s. |  | 0.016 | n.s. | n.s. |
|  | SARAII-70 | 0.016 | n.s. | n.s. |  | n.s. | n.s. | n.s. |
|  | SARAII-30 | 0.004 | n.s. | n.s. |  | n.s. | n.s. | 0.011 |
|  | SARAII-CR | 0.007 | n.s. | n.s. |  | n.s. | n.s. | n.s. |
|  | ST-CR | 0.009 | n.s. | n.s |  | n.s. | n.s. | 0.006 |
| *Prevotellaceae UCG-003^5^* | SARAI-70 |  |  |  |  | n.s. | 0.020 | n.s. |
|  | SARAI-30 |  |  |  |  | 0.015 | n.s. | n.s. |
|  | SARAI-CR |  |  |  |  | 0.005 | n.s. | n.s. |
|  | ST-CR |  |  |  |  | n.s. | 0.021 | n.s. |
| *Prevotellaceae YAB2003 group* | SARAI-30 | 0.012 | n.s. | n.s. |  | n.s. | n.s. | n.s. |
|  | SARAII-30 | 0.016 | n.s. | n.s. |  | n.s. | n.s. | n.s. |
| *Pseudobutyri-vibrio^5^* | SARAI-30 | 0.016 | n.s. | n.s. |  |  |  |  |
|  | SARAI-CR | 0.005 | n.s. | n.s. |  |  |  |  |
|  | SARAII-70 | 0.016 | n.s. | n.s. |  |  |  |  |
|  | SARAII-30 | 0.007 | n.s. | n.s. |  |  |  |  |
|  | SARAII-CR | 0.012 | n.s. | n.s. |  |  | . | . |
|  | ST-CR | 0.007 | n.s. | n.s. |  |  |  |  |
| *Pseudomonas^5^* | SARAI-70 |  |  |  |  | 0.022 | n.s. | n.s. |
|  | SARAI-CR |  |  |  |  | 0.010 | n.s. | n.s. |
| *Rikenellaceae RC9 gut group* | SARAI-30 | 0.012 | n.s. | n.s. |  | n.s. | 0.012 | n.s. |
|  | SARAI-CR | 0.016 | n.s. | n.s. |  | n.s. | 0.009 | n.s. |
|  | SARAII-70 | 0.021 | n.s. | n.s. |  | n.s. | n.s. | n.s. |
|  | SARAII-30 | 0.009 | n.s. | n.s. |  | n.s. | n.s. | n.s. |
|  | SARAII-CR | n.s. | 0.016 | n.s. |  | n.s. | n.s. | n.s. |
|  | ST-CR | 0.007 | n.s. | n.s. |  | n.s. | 0.016 | n.s. |
| *Schwartzia^5^* | SARAI-30 | n.s. | 0.021 | n.s. |  |  |  |  |
|  | SARAII-70 | 0.009 | n.s. | n.s. |  |  |  |  |
| *Selenomonas 1* | SARAI-30 | n.s. | 0.009 | n.s. |  | n.s. | n.s. | n.s. |
| *Sphaerochaeta^5^* | SARAI-70 |  |  |  |  | n.s. | 0.021 | n.s. |
|  | SARAI-CR |  |  |  |  | n.s. | 0.007 | n.s. |
|  | SARAII-70 |  |  |  |  | n.s. | 0.003 | n.s. |
| *Streptococcus* | ST-CR | n.s. | n.s. | n.s. |  | n.s. | 0.021 | n.s. |
| *Treponema 2* | SARAI-70 | n.s. | 0.012 | n.s. |  | n.s. | 0.016 | n.s. |
|  | SARAI-30 | n.s. | 0.005 | n.s. |  | n.s. | 0.012 | n.s. |
|  | SARAI-CR | n.s. | 0.005 | n.s. |  | n.s. | 0.011 | n.s. |
|  | SARAII-CR | n.s. | 0.007 | n.s. |  | n.s. | n.s. | n.s. |
| *Veillonellaceae UCG-001^5^* | SARAI-30 | 0.003 | n.s. | n.s. |  |  |  |  |
|  | SARAI-CR | 0.005 | n.s. | n.s. |  |  |  |  |
|  | SARAII-70 | 0.009 | n.s. | n.s. |  |  |  |  |
|  | SARAII-30 | 0.007 | n.s. | n.s. |  |  |  |  |
|  | SARAII-CR | 0.016 | n.s. | n.s. |  |  |  |  |
|  | ST-CR | 0.007 | n.s. | n.s. |  |  |  |  |

^1^period: CP I = control period I; SARA = SARA period; CP II = control period II

^2^ only genera with significant changes are listed

^3^Treatment groups: SARAI-70 = SARA I buffer, 70% concentrate; SARAI-30 = SARA I buffer, 30%; SARAI-CR = SARA I buffer, changing ratio; SARAII-70 = SARA II buffer, 70% concentrate; SARAII-30 = SARA II buffer, 30% concentrate; SARAII-CR = SARA II buffer, changing ratio; ST-CR = Standard buffer, changing ratio; ST-70 = Standard buffer, 70% concentrate. Only groups with significant changes are displayed.

^4^ n.s. = not significant

^5^ among the 25 most abundant genera for only one of two phases
