# Supplementary material for: PacBio and Illumina MiSeq Amplicon Sequencing Confirm Full Recovery of the Bacterial Community After Subacute Ruminal Acidosis Challenge in the RUSITEC System
Source: Front Microbiol. 2020 Aug 7;11:1813. doi: 10.3389/fmicb.2020.01813 (PMC7426372; doi:10.3389/fmicb.2020.01813)
Supplement: Supplementary file 13 [file Table_6.DOCX]

**Supplementary Table 6. Significant alterations of the 50 most abundant amplicon sequence variants (ASVs) within the solid phase detected by applying the PacBio technique**

|  | | treatment group^1^ | | | | | | | |
| --- | --- | --- | --- | --- | --- | --- | --- | --- | --- |
|  |  | ST-70 | ST-CR | SARA I-70 | SARA I-CR | SARA I-30 | SARA II-70 | SARA II-CR | SARA II-30 |
| ASV – ID ^2^ | period^3^ | *P*-value^4^ | | | | | | | |
| 74_g_*Butyrivibrio_2* | SARA – CP II | n.s. | n.s. | n.s. | n.s. | n.s. | 0.017 | 0.007 | n.s. |
|  | CP I – CP II | n.s. | n.s. | n.s. | n.s. | n.s. | n.s. | 0.024 | 0.012 |
| 4_f_*F082* | CP I – SARA | n.s. | n.s. | n.s. | 0.007 | n.s. | n.s. | n.s. | 0.008 |
| 66_g_*Fibrobacter* | CP I – SARA | n.s. | n.s. | n.s. | n.s. | n.s. | n.s. | n.s. | n.s. |
|  | SARA – CP II | n.s. | n.s. | 0.005 | 0.011 | 0.004 | n.s. | n.s. | 0.012 |
|  | CP I – CP II | n.s. | n.s. | n.s. | n.s. | n.s. | n.s. | n.s. | n.s. |
| 48_g_*Lactobacillus* | SARA – CP II | n.s. | 0.016 | n.s. | n.s. | n.s. | n.s. | n.s. | n.s. |
| 50_g_*Lactobacillus* | CP I – SARA | n.s. | 0.021 | n.s. | n.s. | n.s. | n.s. | n.s. | n.s. |
| 3_s_*Lactobacillus_ amylovorus* | CP I – SARA | n.s. | n.s. | n.s. | 0.016 | n.s. | n.s. | n.s. | n.s. |
| 24_s_*Lactobacillus_ mucosae* | CP I – SARA | n.s. | n.s. | n.s. | n.s. | n.s. | n.s. | 0.009 | n.s. |
|  | SARA – CP II | n.s. | 0.016 | n.s. | n.s. | n.s. | n.s. | n.s. | n.s. |
| 30_g_*Oribacterium* | CP I – SARA | n.s. | n.s. | n.s. | n.s. | 0.021 | n.s. | n.s. | 0.016 |
| 13_g_*Prevotella_1* | SARA – CP II | n.s. | n.s. | n.s. | 0.018 | 0.012 | 0.021 | n.s. | n.s. |
| 25_g_*Prevotella_1* | SARA – CP II | n.s. | 0.012 | n.s. | n.s. | 0.016 | 0.016 | n.s. | n.s. |
| 33_g_*Prevotella_1* | SARA – CP II | n.s. | 0.014 | n.s. | n.s. | 0.012 | n.s. | n.s. | n.s. |
| 36_g_*Prevotella_1* | SARA – CP II | n.s. | n.s. | n.s. | n.s. | 0.019 | n.s. | n.s. | n.s. |
| 41_g_*Prevotella_1* | SARA – CP II | n.s. | n.s. | n.s. | n.s. | 0.012 | 0.009 | n.s. | n.s. |
| 42_g_*Prevotella_1* | SARA – CP II | n.s. | n.s. | n.s. | n.s. | 0.009 | n.s. | n.s. | n.s. |
| 44_g_*Prevotella_1* | SARA – CP II | n.s. | n.s. | n.s. | n.s. | 0.005 | n.s. | n.s. | n.s. |
| 45_g_*Prevotella_1* | SARA – CP II | n.s. | n.s. | n.s. | n.s. | 0.019 | 0.021 | n.s. | n.s. |
| 53_g_*Prevotellaceae_ YAB2003_group* | CP I – SARA | n.s. | n.s. | n.s. | 0.008 | 0.012 | n.s. | n.s. | 0.016 |
| 58_g_ *Pseudobutyrivibrio* | CP I – SARA | n.s. | 0.009 | n.s. | n.s. | 0.009 | n.s. | 0.004 | 0.007 |
| 23_g_*Rikenellaceae_ RC9_gut_group* | CP I – SARA | n.s. | n.s. | n.s. | n.s. | n.s. | n.s. | n.s. | 0.012 |
|  | SARA – CP II | n.s. | n.s. | 0.007 | 0.014 | 0.011 | n.s. | 0.011 | n.s. |
| 32_g_*Rikenellaceae_ RC9_gut_group* | CP I – SARA | n.s. | n.s. | n.s. | 0.015 | 0.022 | n.s. | n.s. | n.s. |
|  | SARA – CP II | n.s. | n.s. | 0.022 | n.s. | n.s. | n.s. | n.s. | n.s. |
| 34_g_*Rikenellaceae_ RC9_gut_group* | CP I – SARA | n.s. | n.s. | n.s. | n.s. | n.s. | 0.005 | n.s. | n.s. |
|  | SARA – CP II | n.s. | n.s. | 0.014 | n.s. | 0.009 | n.s. | 0.006 | 0.015 |
| 39_g_*Rikenellaceae_ RC9_gut_group* | CP I – SARA | n.s. | n.s. | n.s. | 0.009 | 0.008 | 0.017 | n.s. | n.s. |
|  | CP I – CP II | 0.017 | n.s. | n.s. | n.s. | n.s. | n.s. | n.s. | n.s. |

^1^ Treatment groups: SARAI-70 = SARA I buffer, 70% concentrate; SARAI-30 = SARA I buffer, 30%; SARAI-CR = SARA I buffer, changing ratio; SARAII-70 = SARA II buffer, 70% concentrate; SARAII-30 = SARA II buffer, 30%; SARAII-CR = SARA II buffer, changing ratio; ST-CR = Standard buffer, changing ratio; ST-70 = Standard buffer, 70% concentrate

^2^ only ASVs with significant changes are listed; ASVs are labeled with the ASV-number, followed by the lowest classification level (o = order, f = family, g = genus, s = species) and the taxonomic identification

^3^ period: CP I = control period I; SARA = SARA period; CP II = control period II

^4^ n.s. = not significant
