# Supplementary material for: PacBio and Illumina MiSeq Amplicon Sequencing Confirm Full Recovery of the Bacterial Community After Subacute Ruminal Acidosis Challenge in the RUSITEC System
Source: Front Microbiol. 2020 Aug 7;11:1813. doi: 10.3389/fmicb.2020.01813 (PMC7426372; doi:10.3389/fmicb.2020.01813)
Supplement: Supplementary file 14 [file Table_7.DOCX]

**Supplementary Table 7. Significant alterations of the 50 most abundant amplicon sequence variants (ASVs) within the liquid phase detected with PacBio sequencing**

|  | | treatment group^1^ | | | | | | | |
| --- | --- | --- | --- | --- | --- | --- | --- | --- | --- |
|  |  | ST-70 | ST-CR | SARA I-70 | SARA I-CR | SARA I-30 | SARA II-70 | SARA II-CR | SARA II-30 |
| ASV – ID ^2^ | period^3^ | *P*-value^4^ | | | | | | | |
| 79_g_*Bifidobacterium* | CP I – SARA | n.s. | n.s. | n.s. | n.s. | n.s. | 0.012 | n.s. | n.s. |
| 16_g_*CPla-4_termite_group* | CP I – SARA | n.s. | n.s. | 0.013 | n.s. | n.s. | 0.022 | n.s. | 0.021 |
| 60_g_*CPla-4_termite_group* | CP I – SARA | n.s. | n.s. | 0.010 | n.s. | n.s. | n.s. | n.s. | n.s. |
| 4_f_*F082* | CP I – SARA | n.s. | n.s. | n.s. | 0.006 | n.s. | n.s. | n.s. | 0.022 |
|  | SARA – CP II | n.s. | n.s. | 0.015 | n.s. | n.s. | n.s. | n.s. | n.s. |
| 15_f_*F082* | CP I – SARA | n.s. | n.s. | n.s. | 0.007 | n.s. | n.s. | n.s. | 0.019 |
| 47_f_*F082* | SARA – CP II | n.s. | n.s. | n.s. | n.s. | n.s. | n.s. | 0.013 | n.s. |
| 95_f_*F082* | SARA – CP II | n.s. | n.s. | n.s. | n.s. | 0.016 | n.s. | n.s. | n.s. |
| 11_o_ *Izimaplasmatales* | CP I – SARA | n.s. | n.s. | 0.014 | n.s. | 0.016 | 0.007 | n.s. | 0.008 |
| 1_s_*Lactobacillus_ amylovorus* | CP I – SARA | n.s. | n.s. | n.s. | 0.007 | n.s. | n.s. | n.s. | n.s. |
| 56_o_*Mollicutes_ RF39* | CP I – SARA | n.s. | n.s. | n.s. | 0.011 | 0.012 | n.s. | n.s. | n.s. |
|  | CP I – CP II | n.s. | 0.008 | n.s. | n.s. | n.s. | n.s. | n.s. | n.s. |
| 30_g_*Oribacterium* | CP I – SARA | n.s. | n.s. | n.s. | n.s. | n.s. | n.s. | n.s. | 0.013 |
| 9_g_*Prevotella_1* | CP I – CP II | 0.009 | n.s. | n.s. | n.s. | n.s. | n.s. | n.s. | n.s. |
| 18_g_*Prevotella_1* | CP I – CP II | 0.007 | n.s. | n.s. | n.s. | n.s. | n.s. | n.s. | n.s. |
| 19_g_*Prevotella_1* | CP I – CP II | 0.012 | n.s. | n.s. | n.s. | n.s. | n.s. | n.s. | n.s. |
| 40_g_*Prevotella_1* | CP I – SARA | n.s. | n.s. | n.s. | 0.017 | n.s. | n.s. | n.s. | n.s. |
|  | SARA – CP II | n.s. | n.s. | n.s. | n.s. | 0.003 | 0.005 | n.s. | n.s. |
| 13_g_*Prevotella_1* | SARA – CP II | n.s. | n.s. | n.s. | 0.014 | n.s. | n.s. | n.s. | n.s. |
| 25_g_*Prevotella_1* | SARA – CP II | n.s. | n.s. | n.s. | 0.019 | n.s. | 0.012 | n.s. | n.s. |
| 33_g_*Prevotella_1* | SARA – CP II | n.s. | n.s. | n.s. | 0.015 | n.s. | n.s. | n.s. | n.s. |
| 41_g_*Prevotella_1* | CP I – SARA | n.s. | n.s. | n.s. | 0.009 | n.s. | n.s. | n.s. | n.s. |
|  | SARA – CP II | n.s. | n.s. | n.s. | n.s. | 0.020 | n.s. | n.s. | n.s. |
| 43_g_*Prevotella_1* | CP I – SARA | n.s. | n.s. | n.s. | 0.012 | n.s. | n.s. | n.s. | n.s. |
|  | SARA – CP II | n.s. | n.s. | n.s. | n.s. | 0.017 | n.s. | n.s. | n.s. |
| 36_g_*Prevotella_1* | CP I – SARA | n.s. | n.s. | n.s. | n.s. | n.s. | n.s. | 0.024 | n.s. |
|  | SARA – CP II | n.s. | n.s. | n.s. | n.s. | n.s. | n.s. | 0.007 | n.s. |
| 12_g_*Prevotella_1* | CP I – CP II | 0.009 | n.s. | n.s. | n.s. | n.s. | n.s. | n.s. | n.s. |
| 38_g_*Prevotella_7* | CP I – SARA | n.s. | n.s. | 0.020 | n.s. | n.s. | n.s. | n.s. | n.s. |
| 35_g_*Prevotellaceae_UCG-001* | CP I – SARA | n.s. | n.s. | n.s. | n.s. | 0.025 | n.s. | n.s. | n.s. |
|  | SARA – CP II | n.s. | n.s. | n.s. | n.s. | 0.025 | n.s. | n.s. | n.s. |
| 23_g_*Rikenellaceae_ RC9_gut_group* | SARA – CP II | n.s. | n.s. | 0.006 | 0.006 | n.s. | n.s. | n.s. | n.s. |
| 34_g_*Rikenellaceae_ RC9_gut_group* | SARA – CP II | n.s. | n.s. | n.s. | n.s. | n.s. | n.s. | n.s. | 0.006 |
|  | CP I – CP II | n.s. | 0.018 | n.s. | n.s. | n.s. | n.s. | n.s. | n.s. |
| 32_g_*Rikenellaceae_ RC9_gut_group* | CP I – SARA | n.s. | n.s. | n.s. | 0.003 | n.s. | n.s. | n.s. | n.s. |
|  | SARA – CP II | n.s. | n.s. | n.s. | n.s. | 0.012 | n.s. | n.s. | n.s. |
| 29_g_ *Saccharofermentans* | CP I – SARA | n.s. | n.s. | n.s. | n.s. | n.s. | n.s. | n.s. | 0.020 |
| 77_g_*Selenomonas* | CP I – SARA | n.s. | n.s. | n.s. | n.s. | 0.012 | n.s. | n.s. | 0.020 |
| 20_g_*Streptococcus* | SARA – CP II | n.s. | 0.021 | n.s. | n.s. | n.s. | n.s. | n.s. | 0.021 |

^1^ Treatment groups: SARAI-70 = SARA I buffer, 70% concentrate; SARAI-30 = SARA I buffer, 30%; SARAI-CR = SARA I buffer, changing ratio; SARAII-70 = SARA II buffer, 70% concentrate; SARAII-30 = SARA II buffer, 30%; SARAII-CR = SARA II buffer, changing ratio; ST-CR = Standard buffer, changing ratio; ST-70 = Standard buffer, 70% concentrate

^2^ only ASVs with significant changes are listed; ASVs are labeled with the ASV-number, followed by the lowest classification level (o = order, f = family, g = genus, s = species) and the taxonomic identification

^3^ period: CP I = control period I; SARA = SARA period; CP II = control period II

^4^ n.s. = not significant
